# Supplementary material for: Four New Diterpenoids from the South China Sea Soft Coral Sinularia nanolobata and DFT-Based Structure Elucidation
Source: Molecules. 2023 Sep 30;28(19):6892. doi: 10.3390/molecules28196892 (PMC10574229; doi:10.3390/molecules28196892)
Supplement: Supplementary file 1 [file molecules-28-06892-s001.zip › molecules-2617337-supplementary.pdf]

Supporting Information

Four New Diterpenoids from the South China Sea Soft Coral *Sinularia nanolobata* and DFT-Based Structure Elucidation

Dan-Dan Yu [1], Lin-Mao Ke [1], Jiao Liu [2], Song-Wei Li [3], Ming-Zhi Su [4], Li-Gong Yao [2,4], Hui Luo [1,*] and Yue-Wei Guo [2,3,4,*]

[1] College of Pharmacy, Guangdong Medical University, Zhanjiang 524023, China; ddyu@baridd.ac.cn (D.-D.Y.); klm102198@163.com (L.-M.K.)

[2] State Key Laboratory of Drug Research, Shanghai Institute of Materia Medica, Chinese Academy of Sciences, Shanghai 201203, China; 201728012342081@simm.ac.cn (J.L.); yaoligong@simm.ac.cn (L.-G.Y.)

[3] School of Medicine, Shanghai University, Baoshan District, Shanghai 200444, China; simmswli@163.com

[4] Shandong Laboratory of Yantai Drug Discovery, Bohai Rim Advanced Research Institute for Drug Discovery, Yantai 264117, China; smz0310@163.com

* Correspondence: luohui@gdmu.edu.cn (H.L.); ywguo@simm.ac.cn (Y.-W.G.)

Content

1. Original spectra of 1 3

Figure S1a. ^{1}H NMR spectrum (600 MHz) of **1** in $CDCl_3$ 3

Figure S1b. DEPT135/^{13}C NMR spectrum (150 MHz) of **1** in $CDCl_3$ 3

Figure S1c. HSQC spectrum (600 MHz) of **1** in $CDCl_3$ 4

Figure S1d. ^{1}H-^{1}H COSY spectrum of (600 MHz) **1** in $CDCl_3$ 4

Figure S1e. HMBC spectrum (600 MHz) of **1** in $CDCl_3$ 5

Figure S1f. NOESY spectrum (600 MHz) of **1** in $CDCl_3$ 5

Figure S1g. HR-EIMS of **1** 6

Figure S1h. IR spectrum of **1** 6

Figure S1i. ECD and UV spectra of **1** 7

2. Original spectra of 2 8

Figure S2a. ^{1}H NMR spectrum (600 MHz) of **2** in $CDCl_3$ 8

Figure S2b. DEPT135/^{13}C NMR spectrum (150 MHz) of **2** in $CDCl_3$ 8

Figure S2c. HSQC spectrum (600 MHz) of **2** in $CDCl_3$ 9

Figure S2d. ^{1}H-^{1}H COSY spectrum (600 MHz) of **2** in $CDCl_3$ 9

Figure S2e. HMBC spectrum (600 MHz) of **2** in $CDCl_3$ 10

Figure S2f. NOESY spectrum (600 MHz) of **2** in $CDCl_3$ 10

Figure S2g. HR-EIMS of **2** 11

Figure S2h. IR spectrum of **2** 11

Figure S2i. ECD and UV spectra of **2** 12

3. Original spectra of 3 13

Figure S3a. ^{1}H NMR spectrum (600 MHz) of **3** in $CDCl_3$ 13

Figure S3b. DEPT135/^{13}C NMR spectrum (150 MHz) of **3** in $CDCl_3$ 13

Figure S3c. HSQC spectrum (600 MHz) of **3** in $CDCl_3$ 14

Figure S3d. ^{1}H-^{1}H COSY spectrum (600 MHz) of **3** in $CDCl_3$ 14

Figure S3e. HMBC spectrum (600 MHz) of **3** in $CDCl_3$ 15

Figure S3f. NOESY spectrum (600 MHz) of **3** in $CDCl_3$ 15

Figure S3g. HR-EIMS of **3** 16

Figure S3h. IR spectrum of **3** 16

Figure S3i. ECD and UV spectra of **3** 17

4. Original spectra of 4 18

Figure S4a. ^{1}H NMR spectrum (600 MHz) of **4** in $CDCl_3$ 18

Figure S4b. DEPT135/^{13}C NMR spectrum (150 MHz) of **4** in $CDCl_3$ 18

Figure S4c. HSQC spectrum (600 MHz) of **4** in $CDCl_3$ 19

Figure S4d. ^{1}H-^{1}H COSY spectrum (600 MHz) of **4** in $CDCl_3$ 19

Figure S4e. HMBC spectrum (600 MHz) of **4** in $CDCl_3$ 20

Figure S4f. NOESY spectrum (600 MHz) of **4** in $CDCl_3$ **20**
Figure S4g. HR-EIMS of **4**........ **21**
Figure S4h. IR spectrum of **4** **21**
Figure S4i. ECD and UV spectra of **4** **21**
5. QM-NMR calculation and DP4+ analysis of compound 3 .... **22**
Figure S5a. Structures of studied isomers for compound **3** **22**
Figure S5b. DP4+ results obtained using experimental data of **3** *versus* isomers 1 (**3a**) and 2 (**3b**) **22**
6. TDDFT-ECD calculations of compounds 1-4........ **23**
Figure S6a. Experimental ECD curve of **1**, and calculated ECD spectrum of (12*S*)-**1**........ **23**
Figure S6b. Experimental ECD curve of **2**, and calculated ECD spectrum of (11*R*,12*R*)-**2**...... **23**
Figure S6c. Experimental ECD curve of **3**, and calculated ECD spectrum of (4*S*,10*R*,12*R*)-**3** . **24**
Figure S6d. Experimental ECD curve of **4**, and calculated ECD spectrum of (1*S*, 2*R*)-**4** **24**
Figure S6e. Re-optimized conformers of (12*S*)-**1** calculated at the B3LYP/6-311G(d,p) level with IEFPCM solvent model for acetonitrile. **27**
Figure S6f. Re-optimized conformers of (11*R*, 12*R*)-**2** calculated at the B3LYP/6-311G(d,p) level with IEFPCM solvent model for acetonitrile. **30**
Figure S6g. Re-optimized conformers of (4*S*, 11*R*, 12*R*)-**3** calculated at the B3LYP/6-311G(d,p) level with IEFPCM solvent model for acetonitrile. **33**
Figure S6h. Re-optimized conformers of (1*S*, 2*R*)-**4** calculated at the B3LYP/6-311G(d,p) level with IEFPCM solvent model for acetonitrile. **35**

1. Original spectra of 1

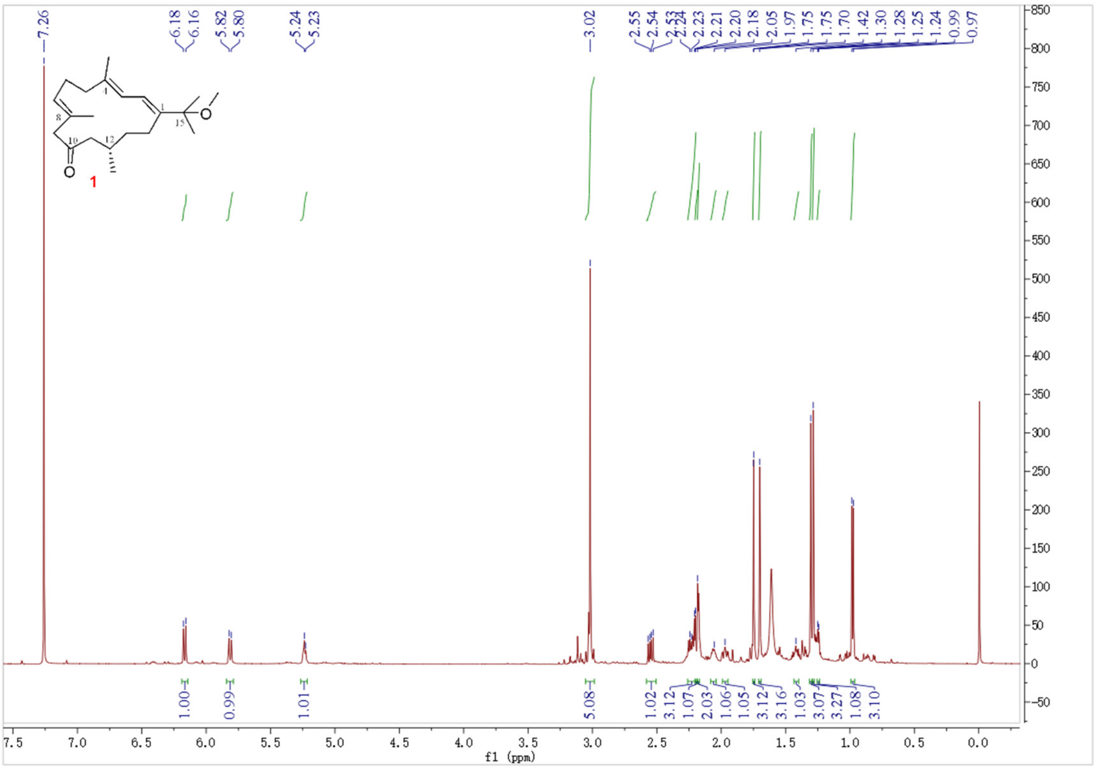

Figure S1a. ^{1}H NMR spectrum (600 MHz) of **1** in $CDCl_3$

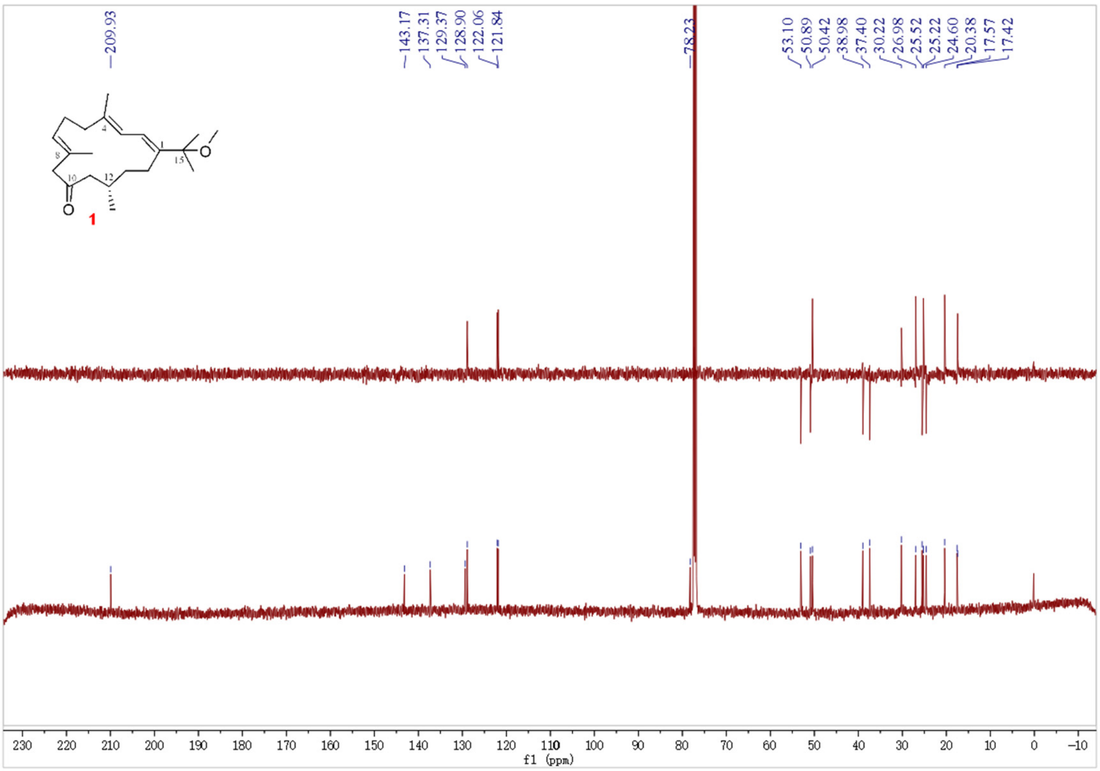

Figure S1b. DEPT135/^{13}C NMR spectrum (150 MHz) of **1** in $CDCl_3$

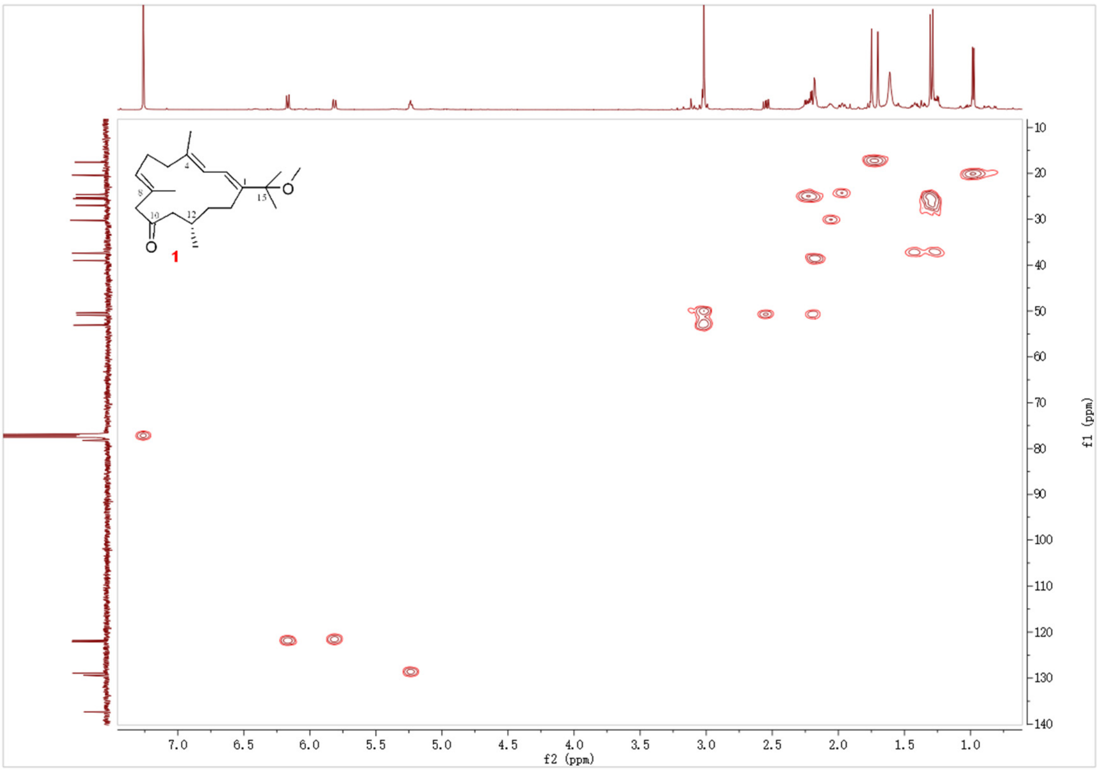

Figure S1c. HSQC spectrum (600 MHz) of **1** in $CDCl_3$

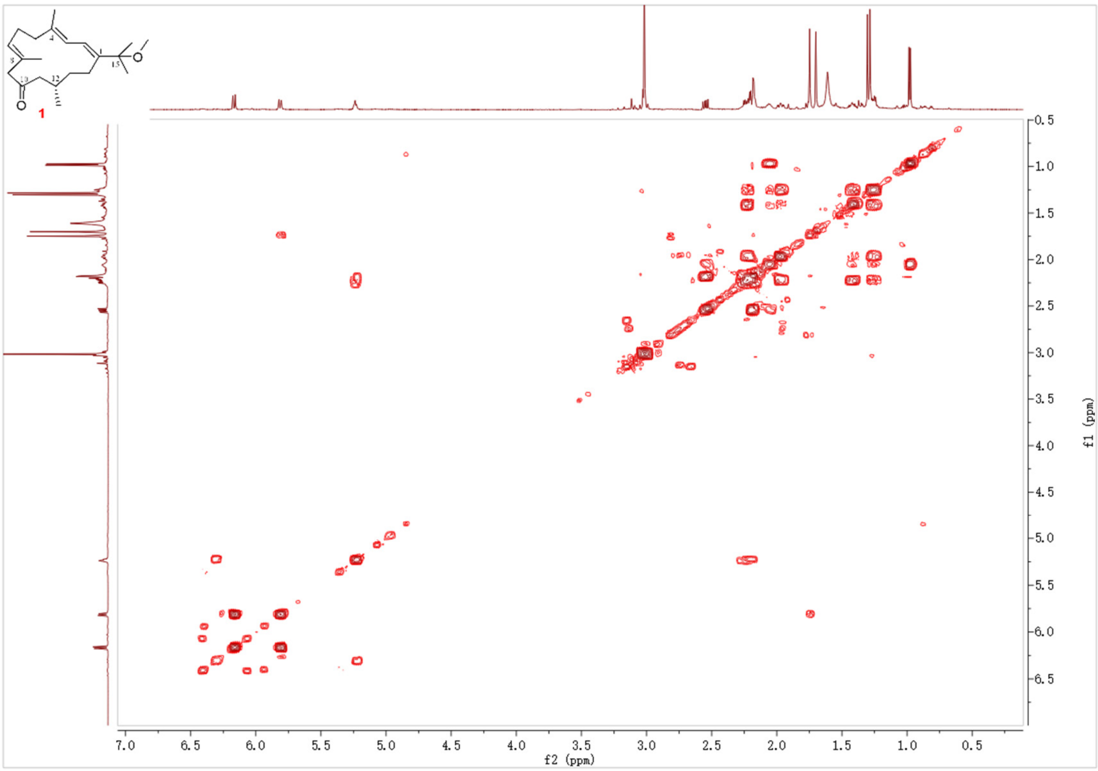

Figure S1d. ^1H-^1H COSY spectrum of (600 MHz) **1** in $CDCl_3$

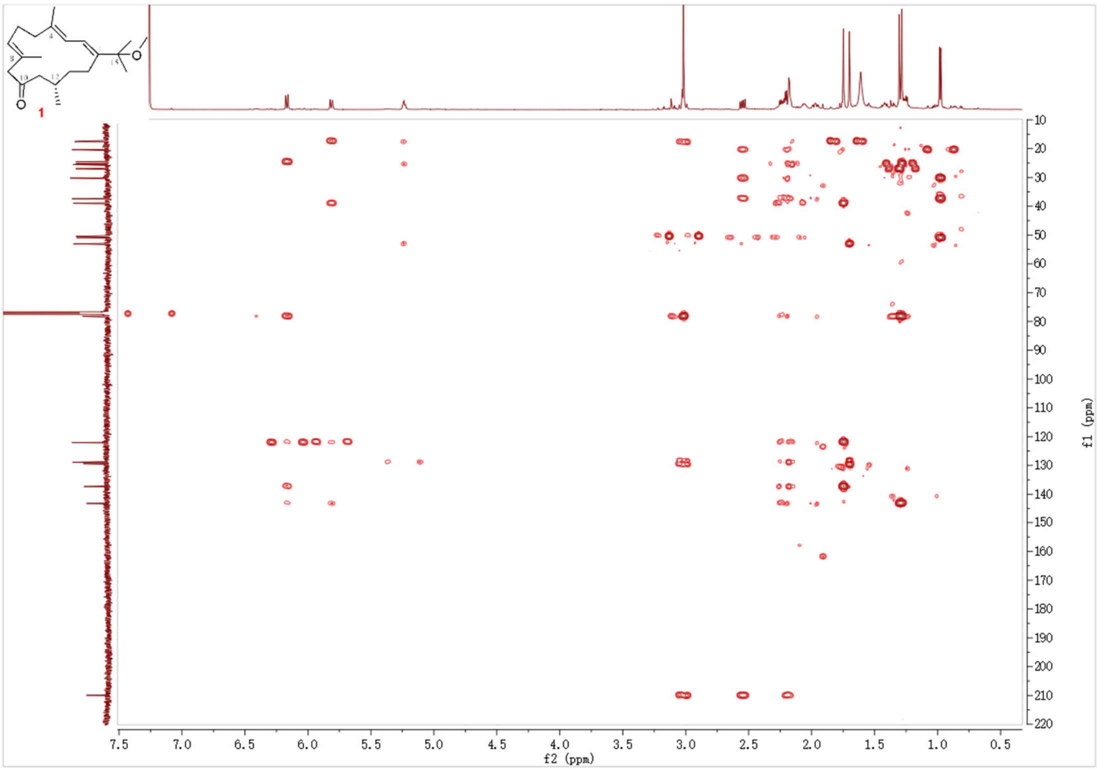

Figure S1e. HMBC spectrum (600 MHz) of **1** in $CDCl_3$

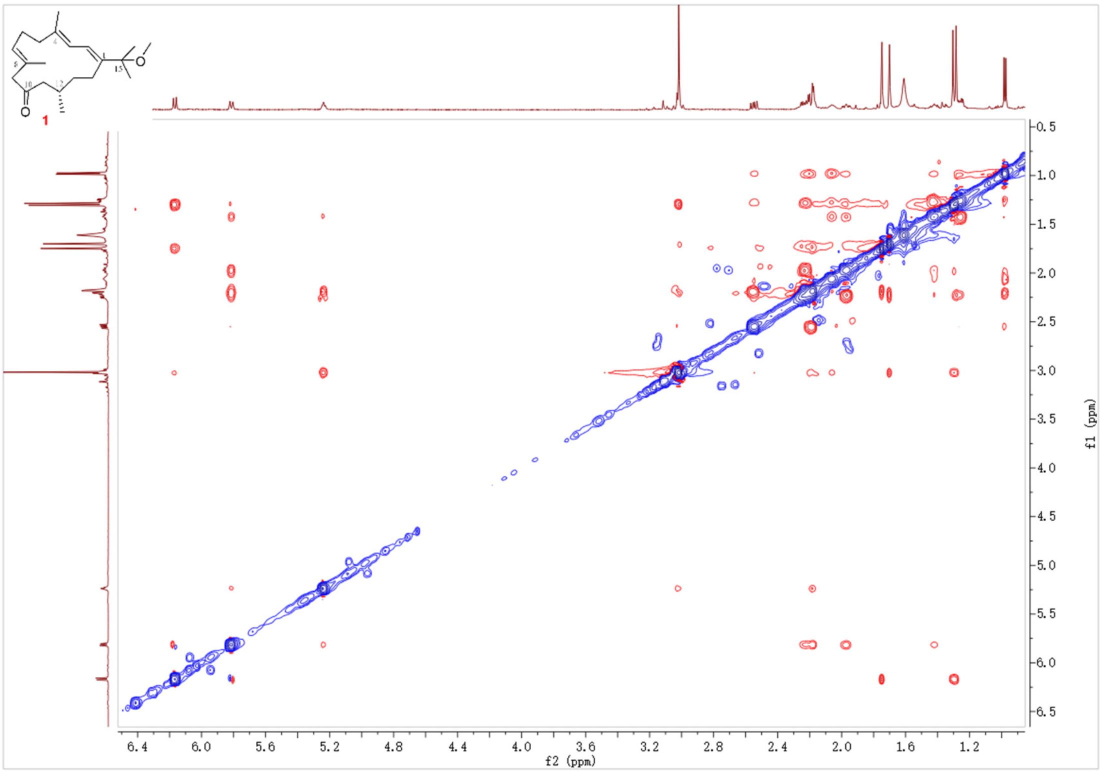

Figure S1f. NOESY spectrum (600 MHz) of **1** in $CDCl_3$

EI202101492_A8-11-212 -c1#8 RT: 1.45
T: + c EI Full ms [49.50-800.50]
m/z= 48-803

m/z	Intensity	Relative	Theo. Mass	Delta (mmu)	RDB equiv.	Composition
53.0360	5522373.0	10.33	53.0386	-2.59	2.5	C_4H_5
59.0521	9796096.0	18.32	59.0491	2.95	0.5	$C_3H_7O_1$
68.0283	5616952.0	10.50	68.0257	2.64	3.0	$C_4H_4O_1$
69.0307	7610319.0	14.23	69.0335	-2.82	2.5	$C_4H_5O_1$
134.0143	8123842.0	15.19	134.0151	-0.84	11.0	$C_{11}H_2$
135.0229	8519424.0	15.93	135.0229	0.01	10.5	$C_{11}H_3$
136.0298	3171483.0	5.93	136.0308	-0.94	10.0	$C_{11}H_4$
139.0175	2208021.0	4.13	139.0178	-0.32	9.5	$C_{10}H_3O_1$
146.0138	2225156.0	4.16	146.0151	-1.26	12.0	$C_{12}H_2$
147.0233	11288576.0	21.11	147.0229	0.39	11.5	$C_{12}H_3$
148.0301	5240100.0	9.80	148.0308	-0.63	11.0	$C_{12}H_4$
149.0028	2527776.0	4.73	149.0022	0.58	11.5	$C_{11}H_1O_1$
149.0388	3180829.0	5.95	149.0386	0.26	10.5	$C_{12}H_5$
150.0102	1820614.0	3.40	150.0100	0.19	11.0	$C_{11}H_2O_1$
151.0183	11781120.0	22.03	151.0178	0.48	10.5	$C_{11}H_3O_1$
152.0261	16571904.0	30.98	152.0257	0.45	10.0	$C_{11}H_4O_1$
153.0317	3542740.0	6.62	153.0335	-1.77	9.5	$C_{11}H_5O_1$
169.0091	1316725.0	2.46	169.0073	1.81	14.5	$C_{14}H_1$
190.0433	1216805.0	2.28	190.0413	1.96	12.0	$C_{14}H_6O_1$
219.1761	1700920.0	3.18	219.1743	1.79	4.5	$C_{15}H_{23}O_1$
243.1728	4137424.0	7.74	243.1743	-1.54	6.5	$C_{17}H_{23}O_1$
244.1794	1968570.0	3.68	244.1822	-2.79	6.0	$C_{17}H_{24}O_1$
253.1939	2065724.0	3.86	253.1951	-1.15	7.5	$C_{19}H_{25}$
268.2195	3925759.0	7.34	268.2186	0.95	7.0	$C_{20}H_{28}$
271.2059	3956501.0	7.40	271.2056	0.25	6.5	$C_{19}H_{27}O_1$
286.2271	18204672.0	34.04	286.2291	-2.03	6.0	$C_{20}H_{30}O_1$
303.2315	13577216.0	25.39	303.2319	-0.32	5.5	$C_{20}H_{31}O_2$
318.2554	1773309.0	3.32	318.2553	0.05	5.0	$C_{21}H_{34}O_2$

Figure S1g. HR-EIMS of **1**

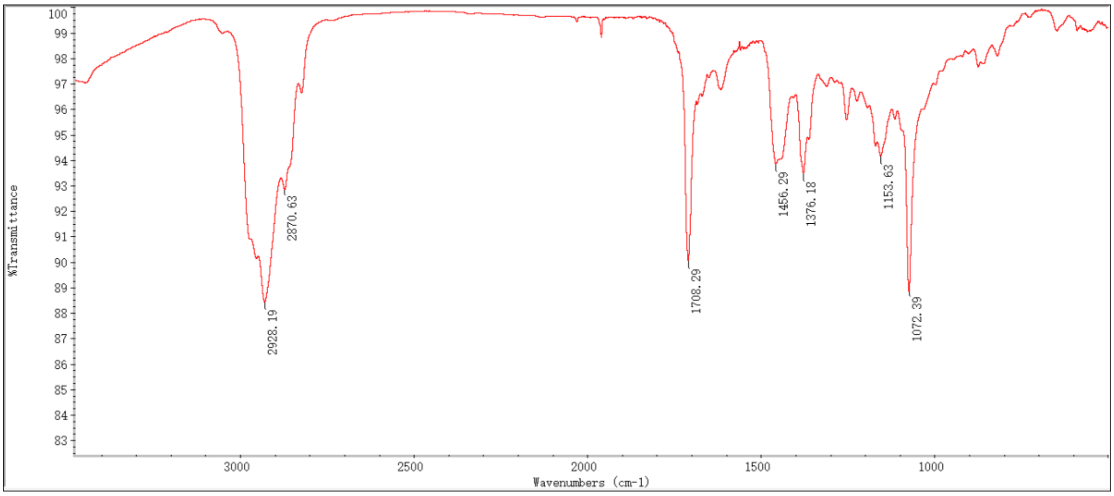

Figure S1h. IR spectrum of **1**

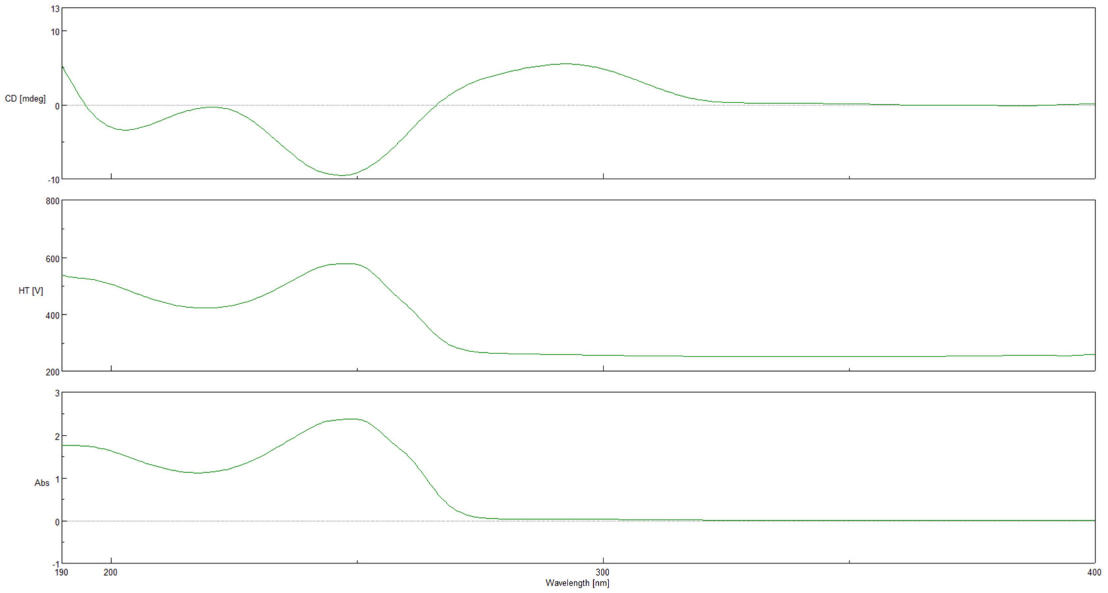

Figure S2i. ECD and UV spectra of **1**

2. Original spectra of 2

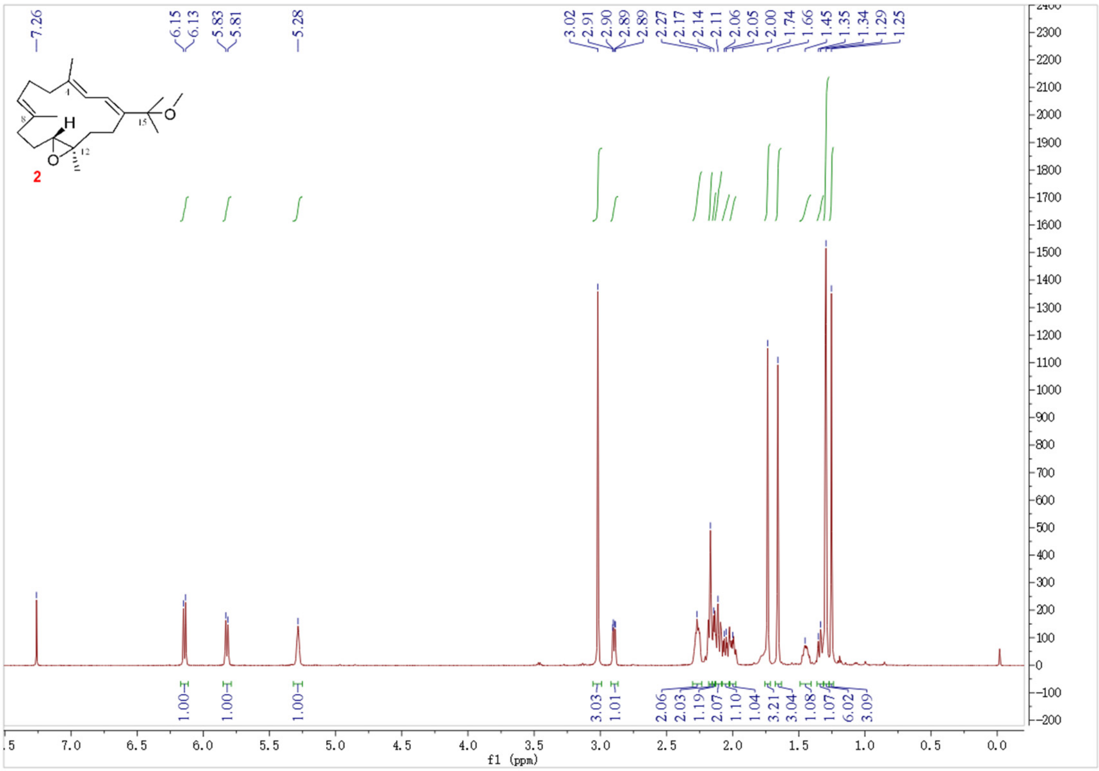

Figure S2a. ^{1}H NMR spectrum (600 MHz) of **2** in $CDCl_3$

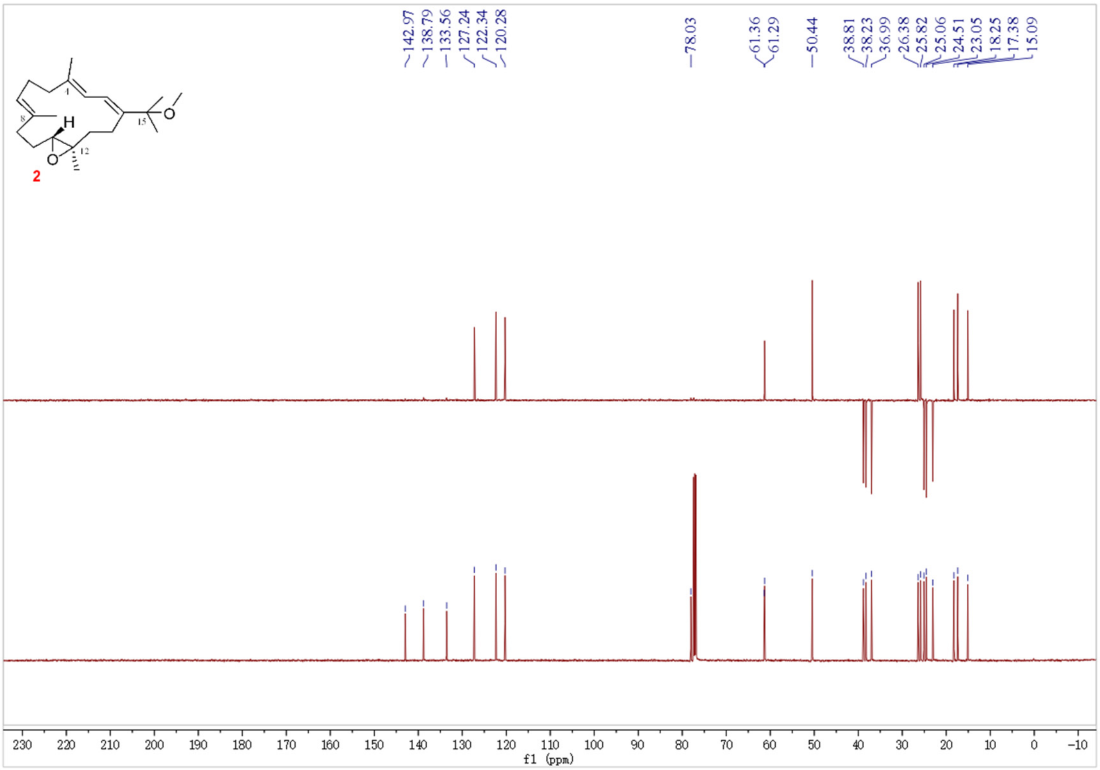

Figure S2b. DEPT135/^{13}C NMR spectrum (150 MHz) of **2** in $CDCl_3$

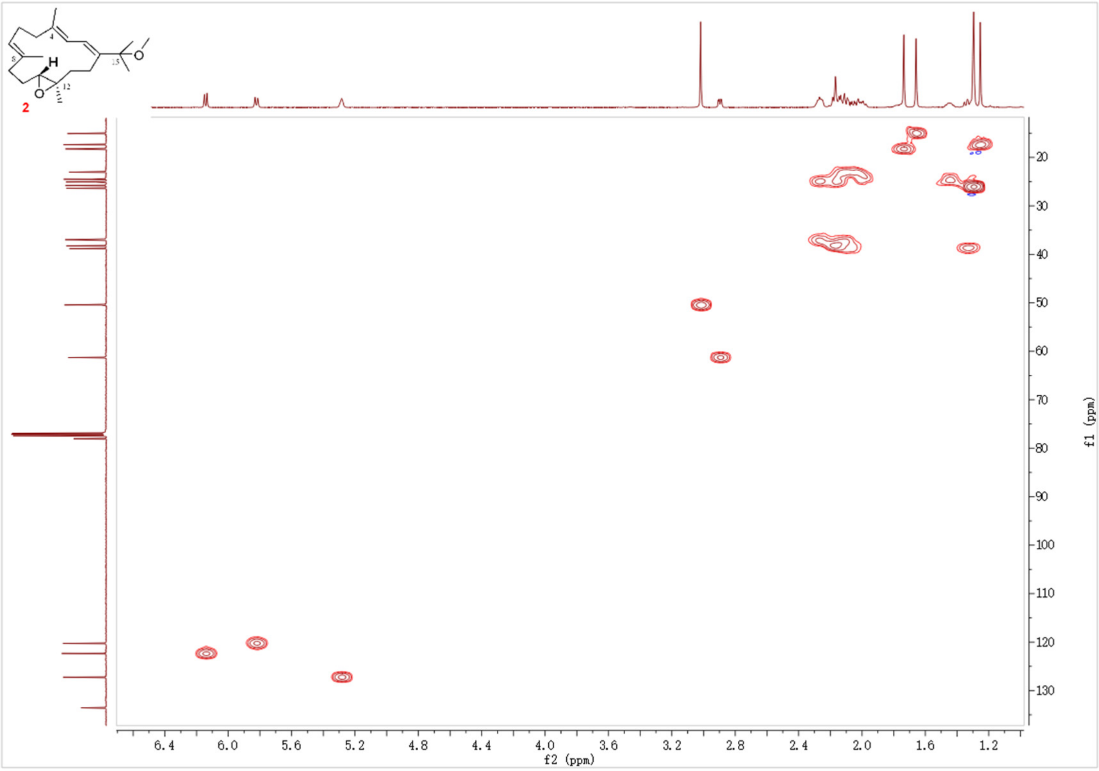

Figure S2c. HSQC spectrum (600 MHz) of **2** in $CDCl_3$

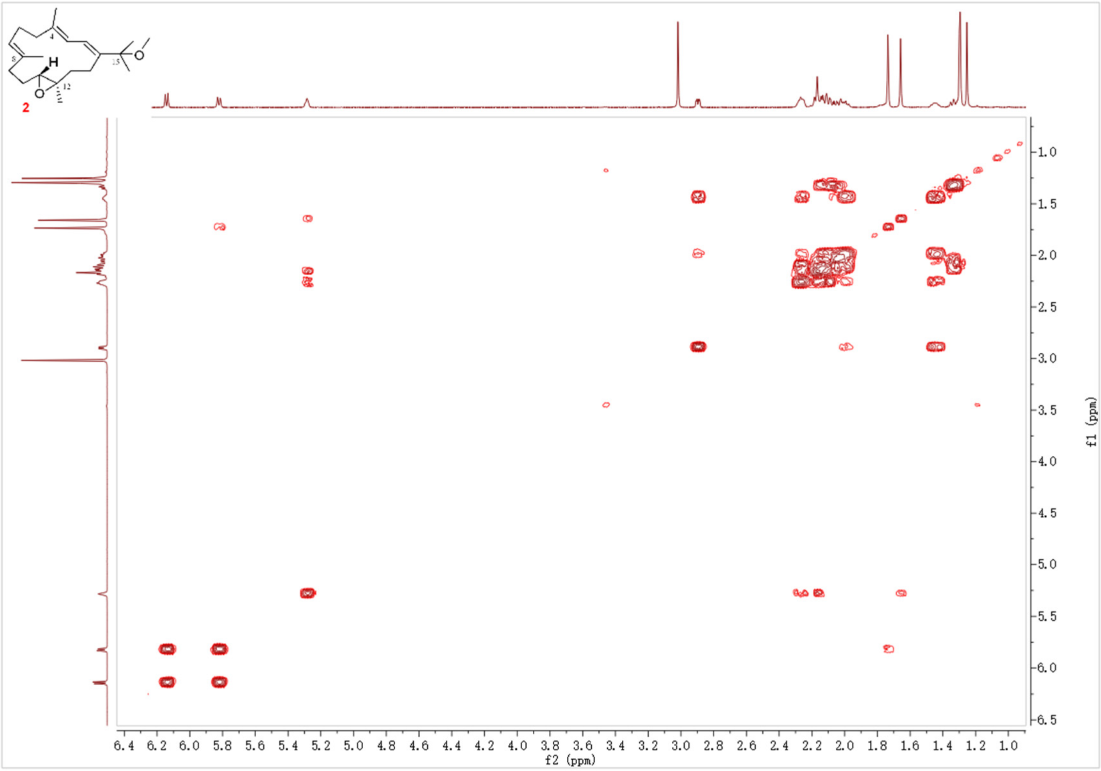

Figure S2d. ^1H-^1H COSY spectrum (600 MHz) of **2** in $CDCl_3$

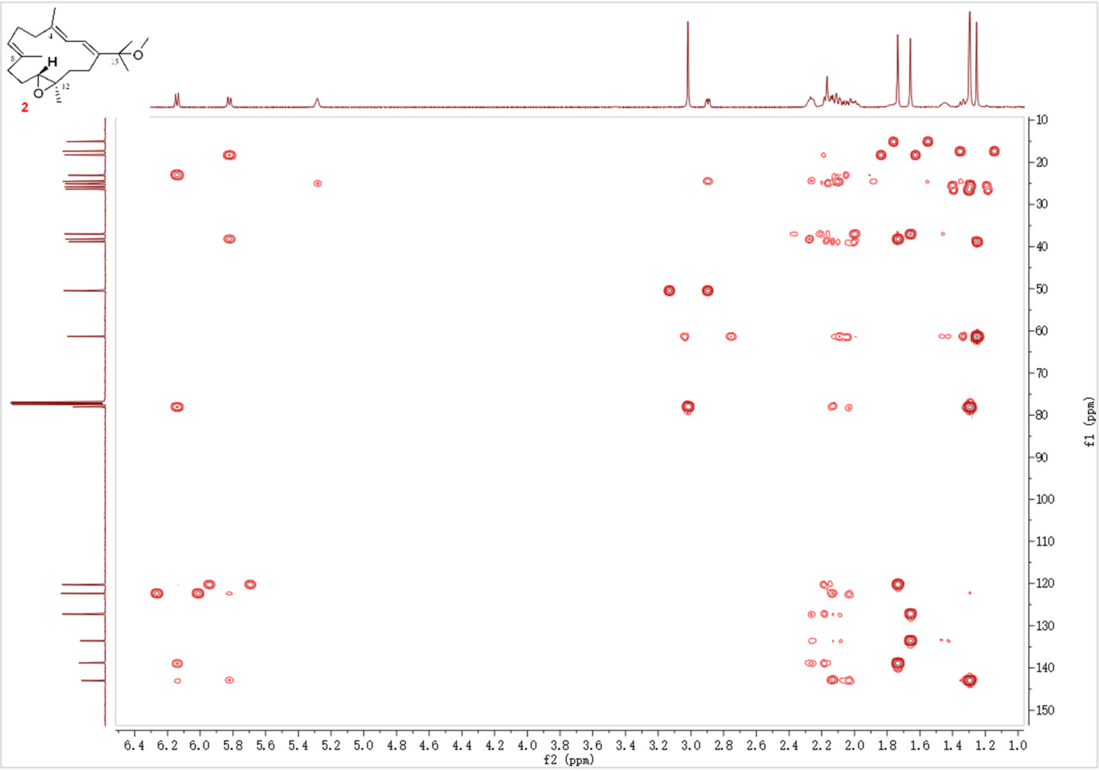

Figure S2e. HMBC spectrum (600 MHz) of **2** in $CDCl_3$

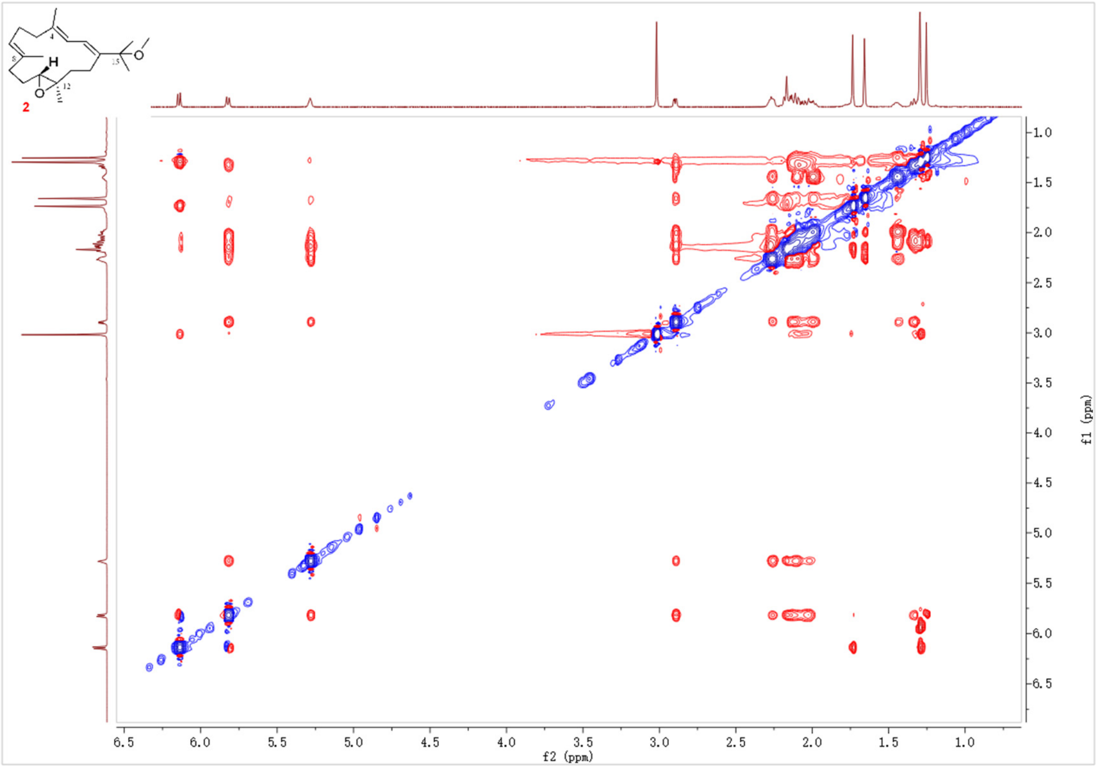

Figure S2f. NOESY spectrum (600 MHz) of **2** in $CDCl_3$

EI202101481_A8-11-214-c1#11 RT: 2.07
T: + c EI Full ms [49.50-800.50]
m/z= 48-803

m/z	Intensity	Relative	Theo. Mass	Delta (mmu)	RDB equiv.	Composition
255.2090	149446.0	0.24	255.2107	-1.72	6.5	$C_{19}H_{27}$
257.1903	301738.0	0.49	257.1900	0.32	6.5	$C_{18}H_{25}O_{1}$
257.2262	272608.0	0.44	257.2264	-0.17	5.5	$C_{19}H_{29}$
258.1969	224007.0	0.36	258.1978	-0.95	6.0	$C_{18}H_{26}O_{1}$
258.2335	295367.0	0.48	258.2342	-0.72	5.0	$C_{19}H_{30}$
259.2037	107573.0	0.18	259.2056	-1.95	5.5	$C_{18}H_{27}O_{1}$
261.1861	47859.0	0.08	261.1849	1.15	5.5	$C_{17}H_{25}O_{2}$
263.2010	76960.0	0.13	263.2006	0.43	4.5	$C_{17}H_{27}O_{2}$
266.2027	28715.0	0.05	266.2029	-0.19	8.0	$C_{20}H_{26}$
267.2105	34198.0	0.06	267.2107	-0.27	7.5	$C_{20}H_{27}$
268.2183	846302.0	1.38	268.2186	-0.29	7.0	$C_{20}H_{28}$
269.1900	46052.0	0.08	269.1900	-0.03	7.5	$C_{19}H_{25}O_{1}$
269.2245	386019.0	0.63	269.2264	-1.88	6.5	$C_{20}H_{29}$
270.2333	173569.0	0.28	270.2342	-0.87	6.0	$C_{20}H_{30}$
271.2058	2512297.0	4.09	271.2056	0.17	6.5	$C_{19}H_{27}O_{1}$
275.2003	119782.0	0.20	275.2006	-0.30	5.5	$C_{18}H_{27}O_{2}$
275.2384	41162.0	0.07	275.2369	1.42	4.5	$C_{19}H_{31}O_{1}$
284.2128	86799.0	0.14	284.2135	-0.69	7.0	$C_{20}H_{28}O_{1}$
285.2211	281053.0	0.46	285.2213	-0.22	6.5	$C_{20}H_{29}O_{1}$
286.2285	5564416.0	9.06	286.2291	-0.58	6.0	$C_{20}H_{30}O_{1}$
287.1997	64010.0	0.10	287.2006	-0.85	6.5	$C_{19}H_{27}O_{2}$
289.2160	28182.0	0.05	289.2162	-0.19	5.5	$C_{19}H_{29}O_{2}$
290.2240	148913.0	0.24	290.2240	-0.03	5.0	$C_{19}H_{30}O_{2}$
300.2442	58261.0	0.09	300.2448	-0.60	6.0	$C_{21}H_{32}O_{1}$
302.2213	30523.0	0.05	302.2240	-2.75	6.0	$C_{20}H_{30}O_{2}$
303.2314	2217226.0	3.61	303.2319	-0.43	5.5	$C_{20}H_{31}O_{2}$
318.2558	224807.0	0.37	318.2553	0.45	5.0	$C_{21}H_{34}O_{2}$

Figure S2g. HR-EIMS of **2**

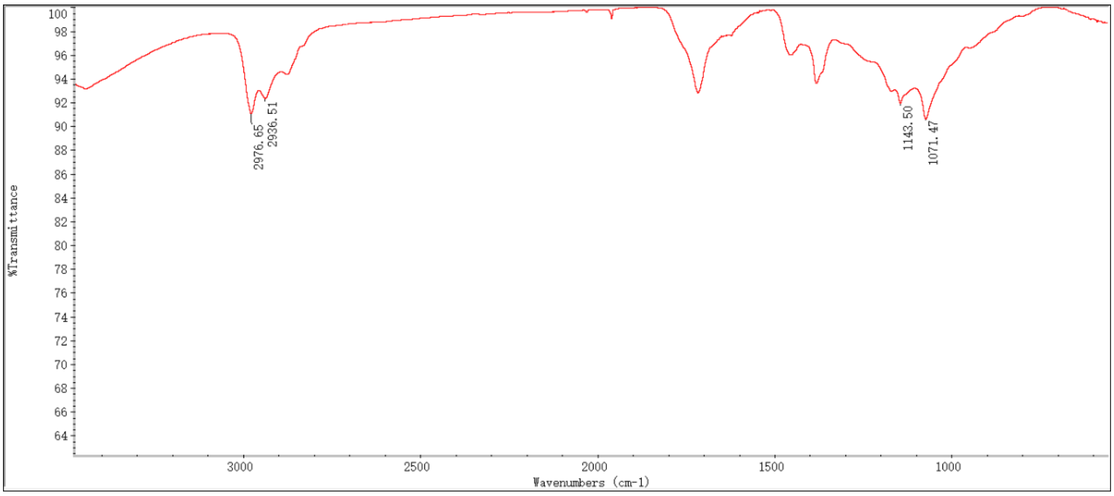

Figure S2h. IR spectrum of **2**

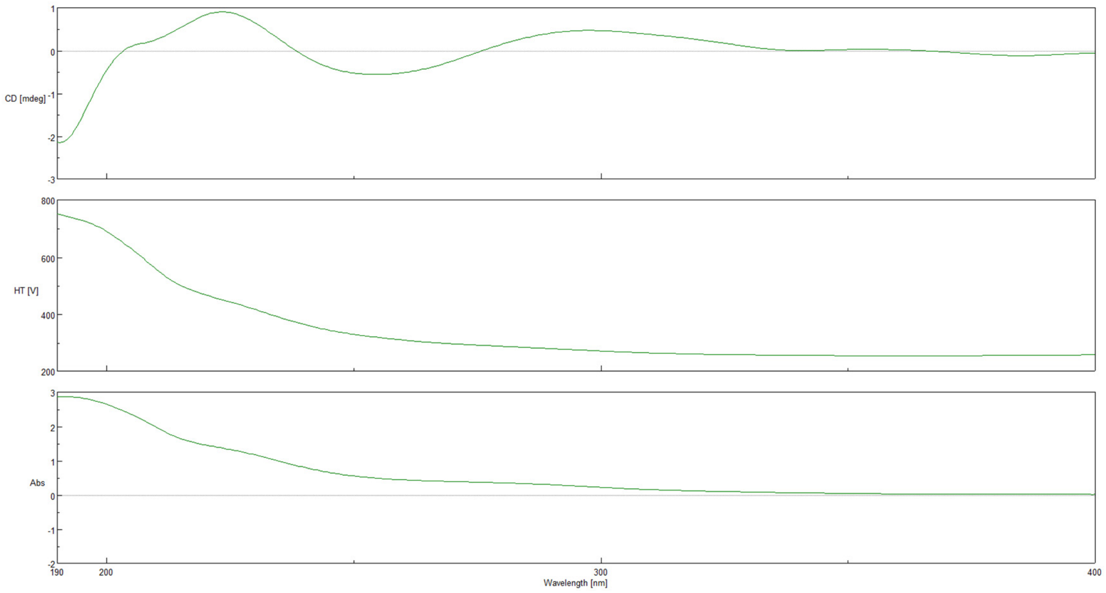

Figure S2i. ECD and UV spectra of **2**

3. Original spectra of 3

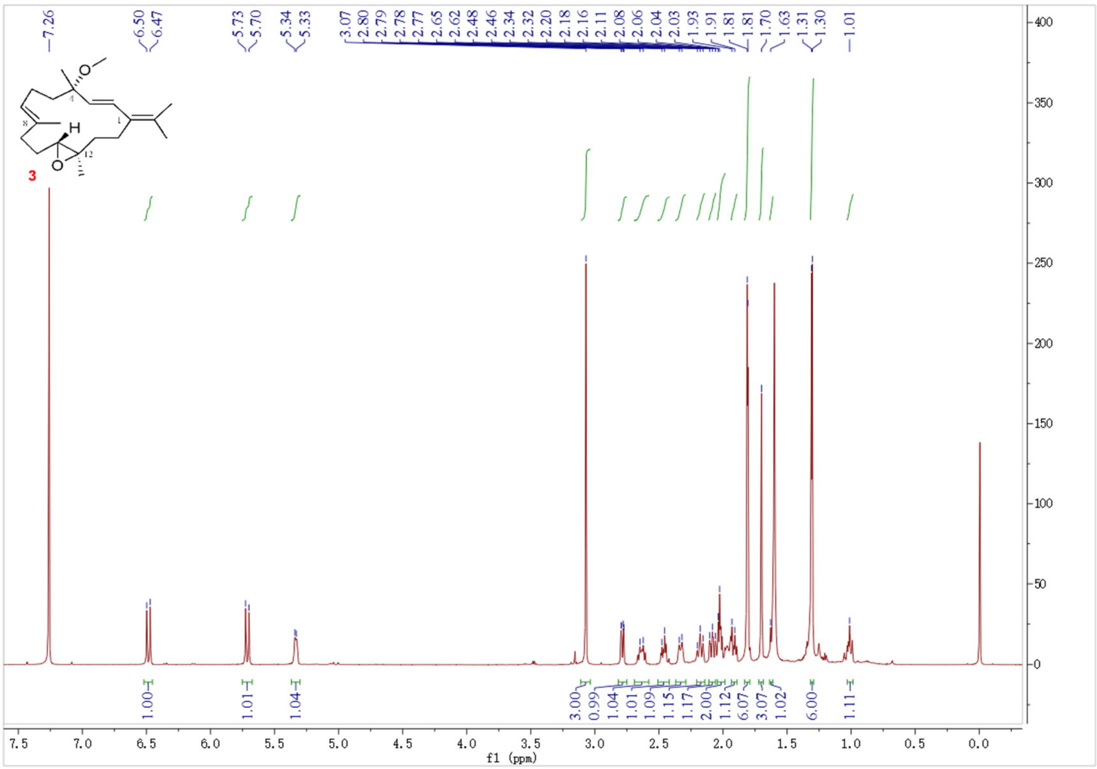

Figure S3a. ^{1}H NMR spectrum (600 MHz) of **3** in $CDCl_3$

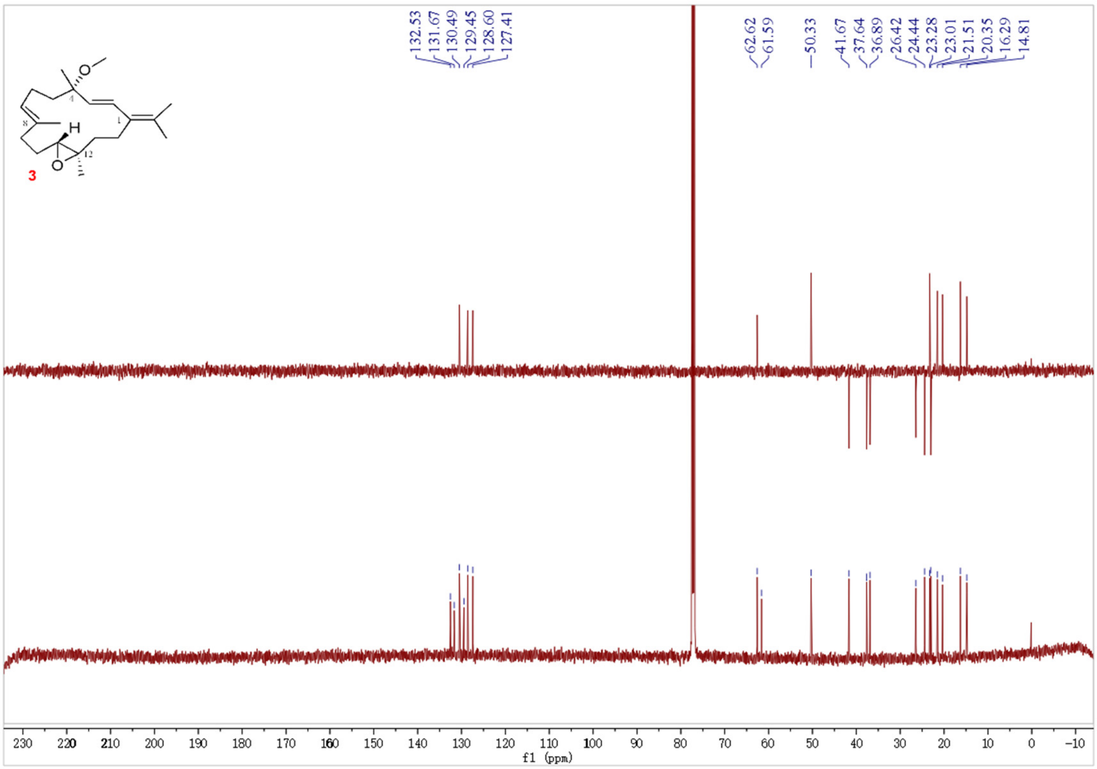

Figure S3b. DEPT135/^{13}C NMR spectrum (150 MHz) of **3** in $CDCl_3$

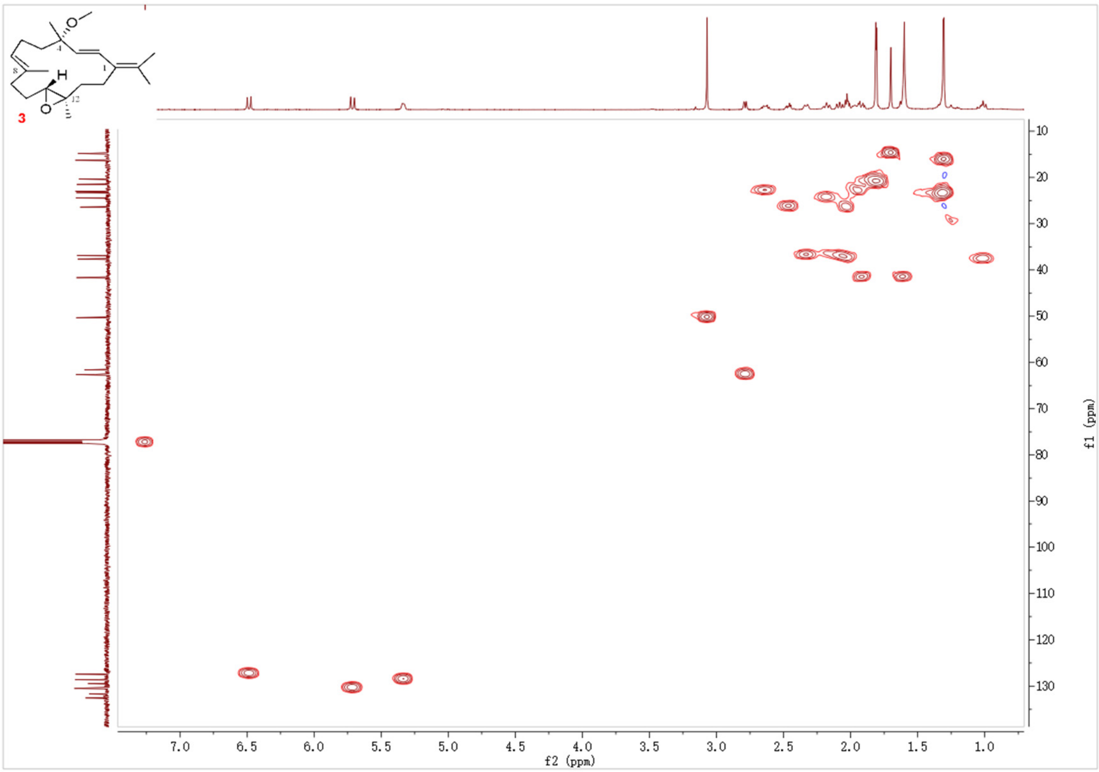

Figure S3c. HSQC spectrum (600 MHz) of **3** in $CDCl_3$

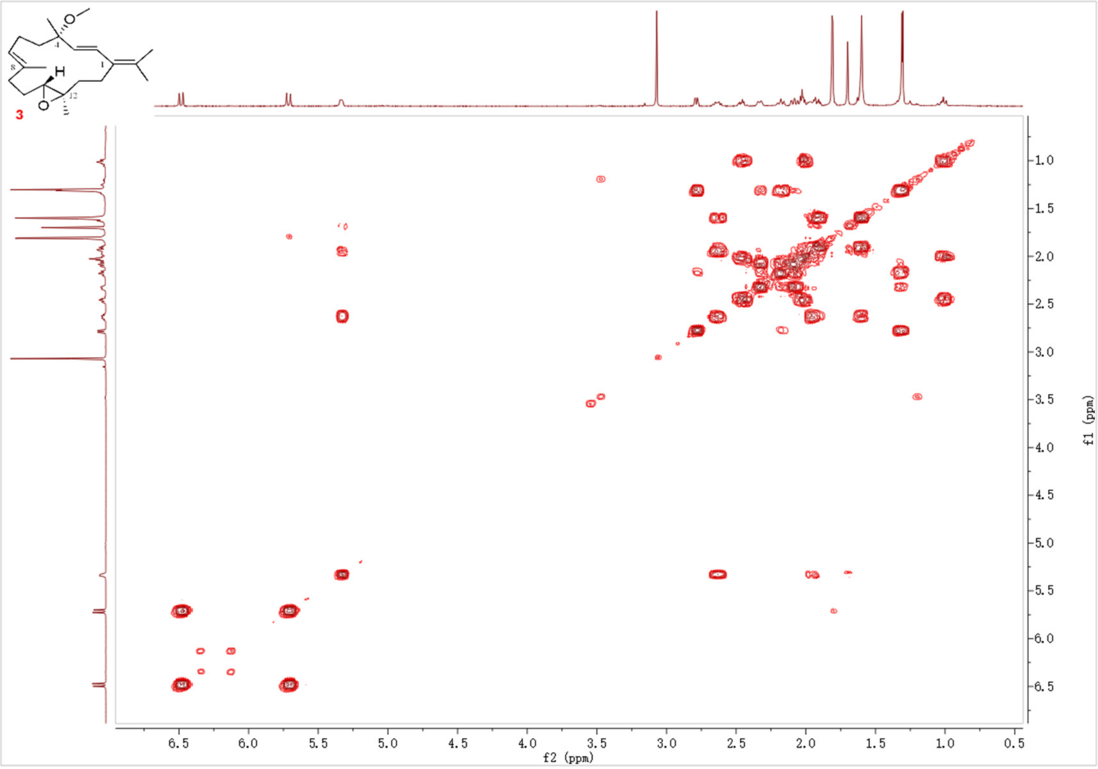

Figure S3d. ^{1}H-^{1}H COSY spectrum (600 MHz) of **3** in $CDCl_3$

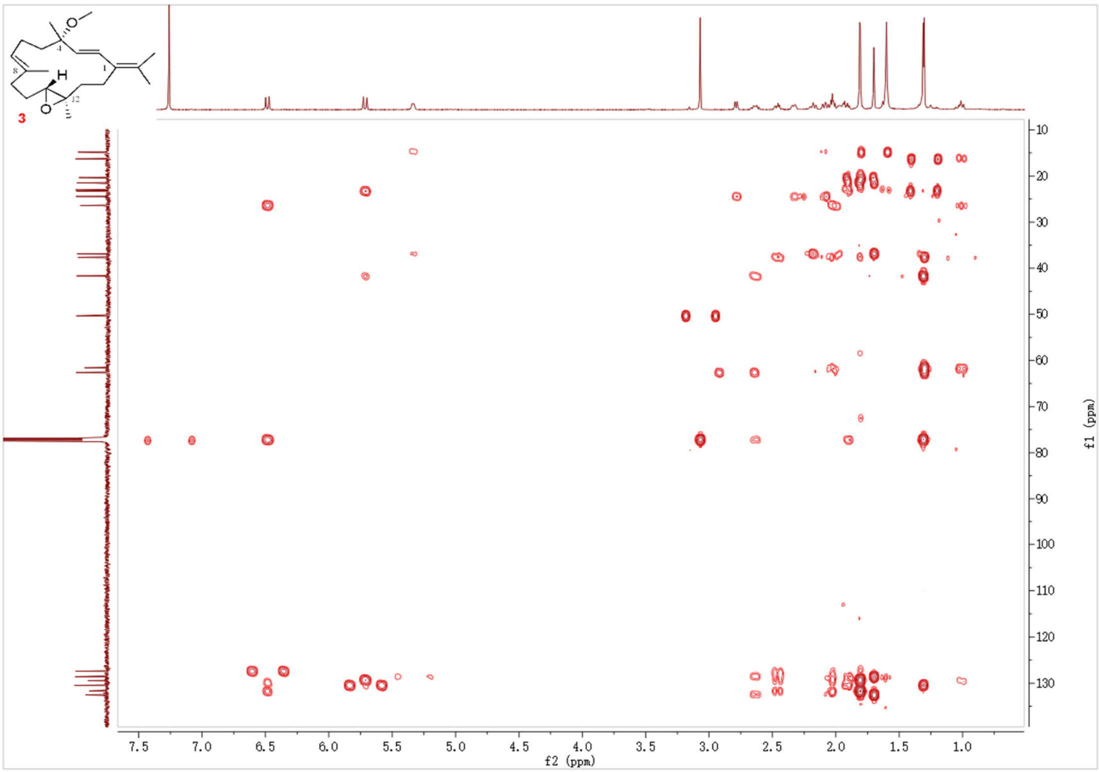

Figure S3e. HMBC spectrum (600 MHz) of **3** in $CDCl_3$

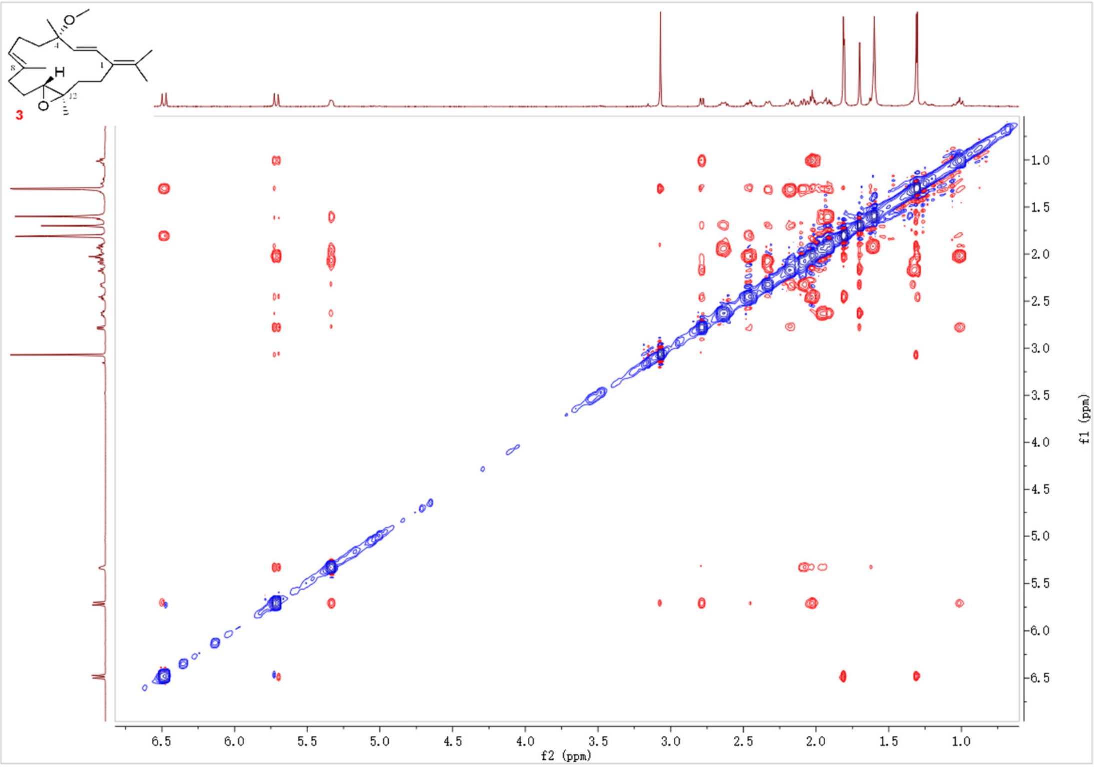

Figure S3f. NOESY spectrum (600 MHz) of **3** in $CDCl_3$

EI202101494_A8-11-215 -c1#7 RT: 1.24
T: + c EI Full ms [49.50-800.50]
m/z= 48-803

m/z	Intensity	Relative	Theo. Mass	Delta (mmu)	RDB equiv.	Composition
206.1692	299536.0	3.13	206.1665	2.68	4.0	$C_{14}H_{22}O_{1}$
216.1891	239132.0	2.50	216.1873	1.84	5.0	$C_{16}H_{24}$
218.1668	303319.0	3.17	218.1665	0.24	5.0	$C_{15}H_{22}O_{1}$
219.1736	238083.0	2.49	219.1743	-0.76	4.5	$C_{15}H_{23}O_{1}$
221.1552	246921.0	2.58	221.1536	1.59	4.5	$C_{14}H_{21}O_{2}$
225.1638	575387.0	6.01	225.1638	0.05	7.5	$C_{17}H_{21}$
226.1700	261641.0	2.73	226.1716	-1.63	7.0	$C_{17}H_{22}$
227.1788	394432.0	4.12	227.1794	-0.61	6.5	$C_{17}H_{23}$
228.1864	545217.0	5.70	228.1873	-0.85	6.0	$C_{17}H_{24}$
229.1592	351419.0	3.67	229.1587	0.54	6.5	$C_{16}H_{21}O_{1}$
229.1947	334569.0	3.50	229.1951	-0.38	5.5	$C_{17}H_{25}$
230.1653	176917.0	1.85	230.1665	-1.22	6.0	$C_{16}H_{22}O_{1}$
231.1743	485863.0	5.08	231.1743	-0.08	5.5	$C_{16}H_{23}O_{1}$
233.1903	141597.0	1.48	233.1900	0.34	4.5	$C_{16}H_{25}O_{1}$
235.1691	652449.0	6.82	235.1693	-0.18	4.5	$C_{15}H_{23}O_{2}$
239.1790	171767.0	1.79	239.1794	-0.39	7.5	$C_{18}H_{23}$
240.1871	165504.0	1.73	240.1873	-0.19	7.0	$C_{18}H_{24}$
243.1748	790804.0	8.26	243.1743	0.49	6.5	$C_{17}H_{23}O_{1}$
243.2110	395481.0	4.13	243.2107	0.30	5.5	$C_{18}H_{27}$
244.1803	256967.0	2.68	244.1822	-1.85	6.0	$C_{17}H_{24}O_{1}$
245.1884	275120.0	2.87	245.1900	-1.59	5.5	$C_{17}H_{25}O_{1}$
253.1959	1050251.0	10.97	253.1951	0.81	7.5	$C_{19}H_{25}$
257.1909	249846.0	2.61	257.1900	0.89	6.5	$C_{18}H_{25}O_{1}$
268.2189	315463.0	3.30	268.2186	0.37	7.0	$C_{20}H_{28}$
269.2262	161117.0	1.68	269.2264	-0.14	6.5	$C_{20}H_{29}$
271.2059	1014836.0	10.60	271.2056	0.28	6.5	$C_{19}H_{27}O_{1}$
285.2215	244696.0	2.56	285.2213	0.20	6.5	$C_{20}H_{29}O_{1}$
286.2292	1024691.0	10.70	286.2291	0.06	6.0	$C_{20}H_{30}O_{1}$
287.2347	368363.0	3.85	287.2369	-2.20	5.5	$C_{20}H_{31}O_{1}$
303.2312	767119.0	8.01	303.2319	-0.70	5.5	$C_{20}H_{31}O_{2}$
318.2566	174565.0	1.82	318.2553	1.24	5.0	$C_{21}H_{34}O_{2}$

Figure S3g. HR-EIMS of **3**

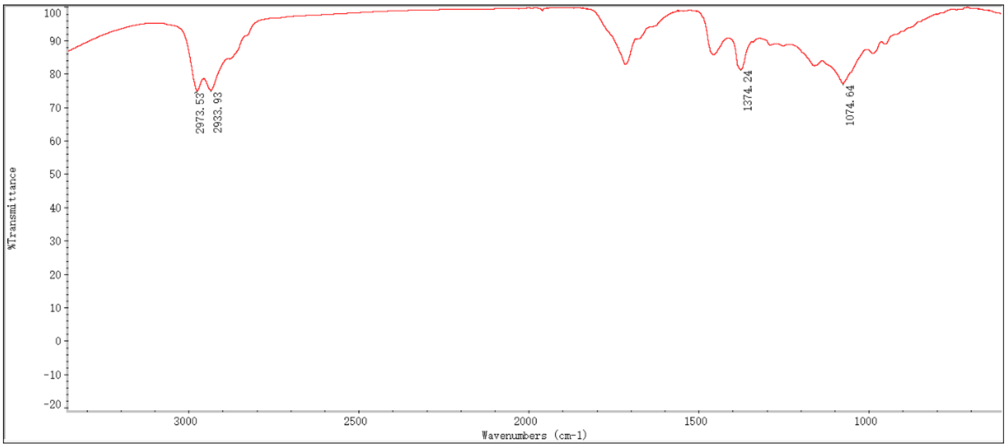

Figure S3h. IR spectrum of **3**

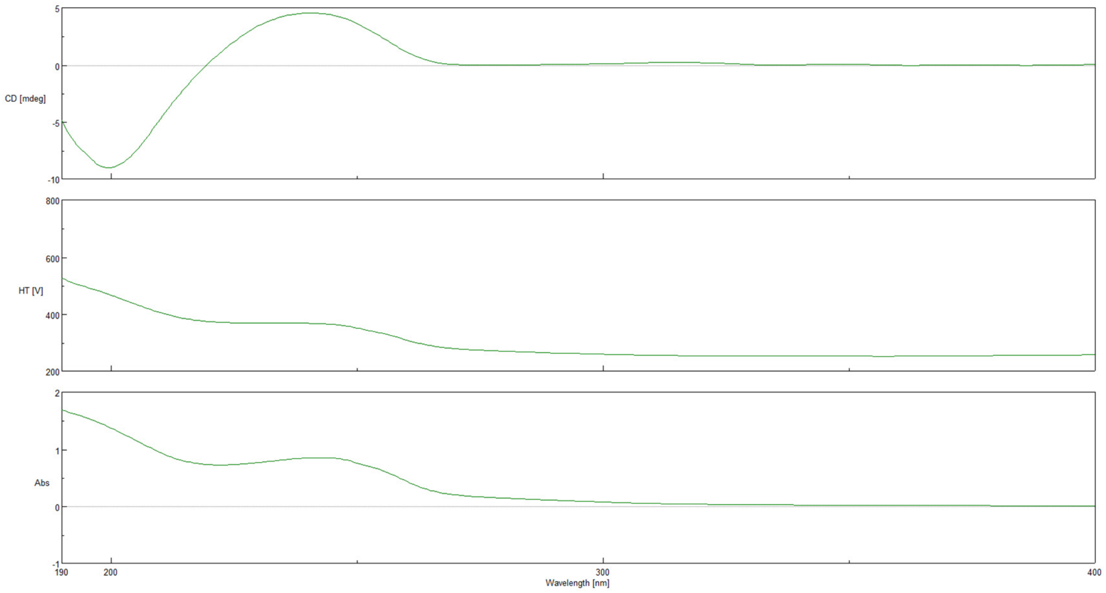

Figure S3i. ECD and UV spectra of **3**

4. Original spectra of 4

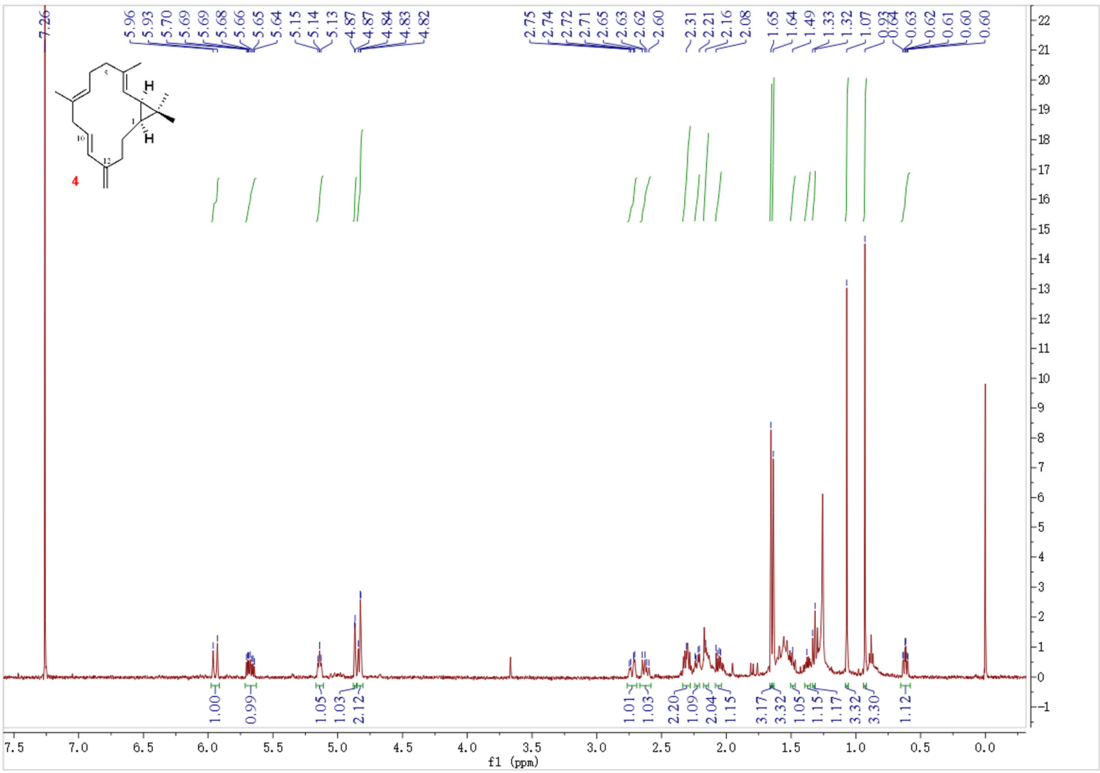

Figure S4a. ^{1}H NMR spectrum (600 MHz) of **4** in $CDCl_3$

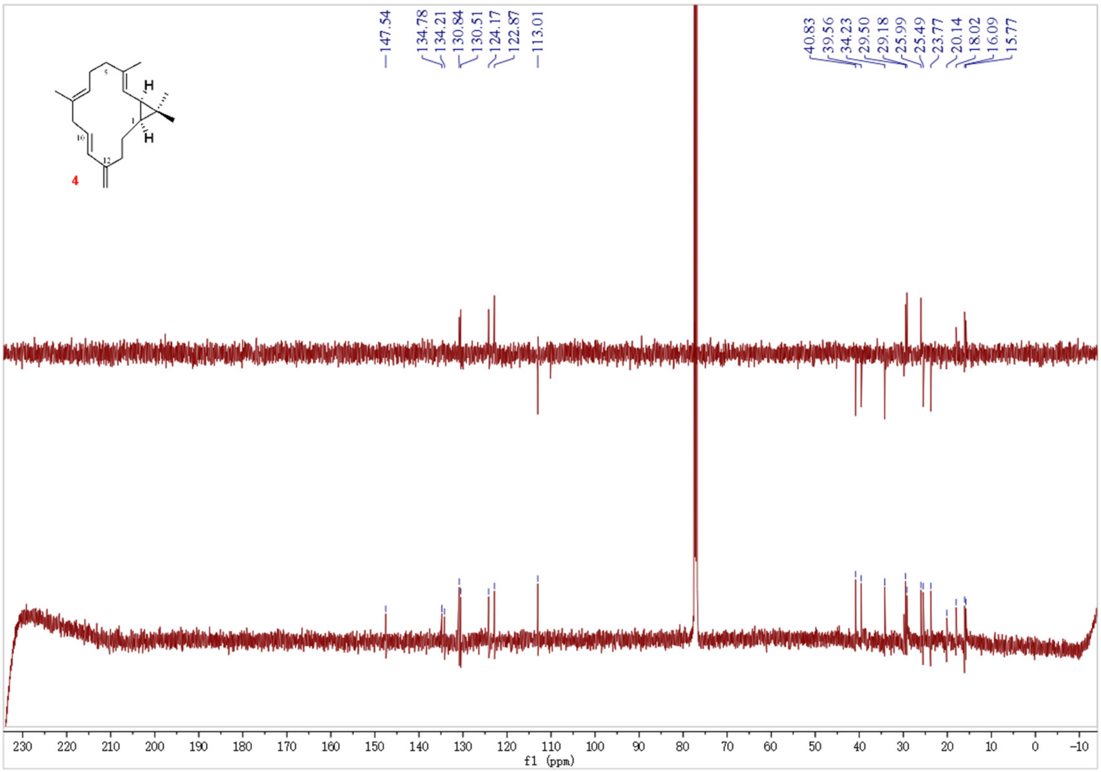

Figure S4b. DEPT135/^{13}C NMR spectrum (150 MHz) of **4** in $CDCl_3$

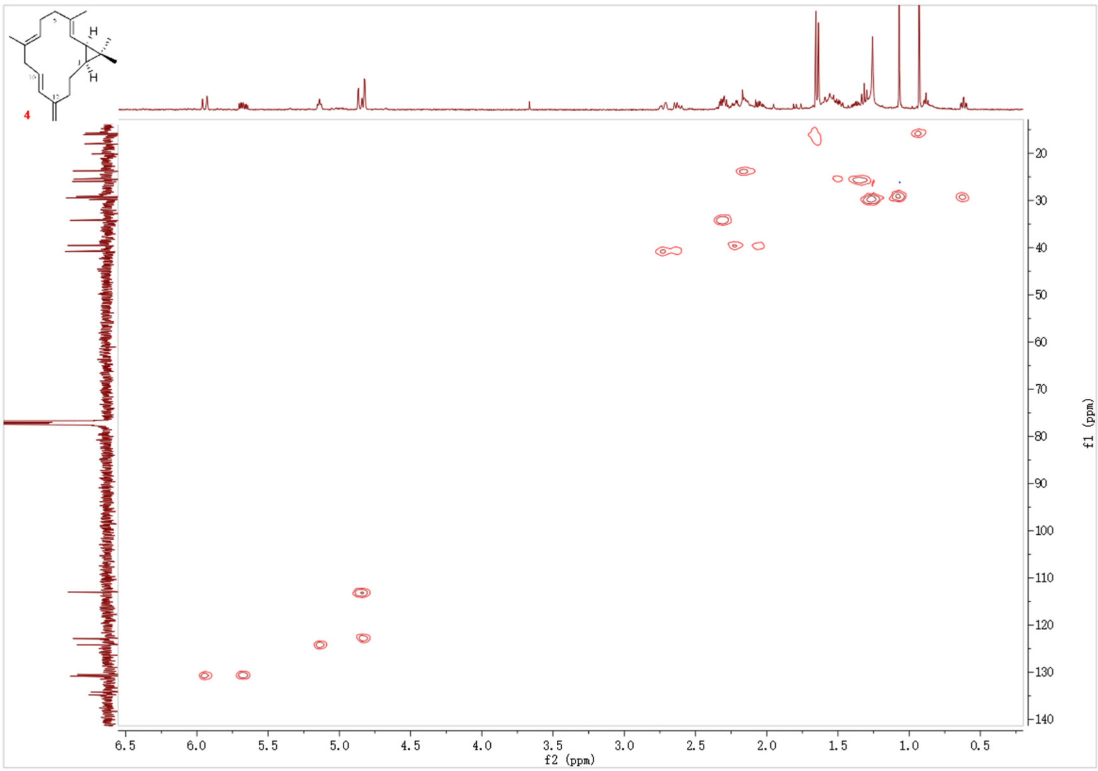

Figure S4c. HSQC spectrum (600 MHz) of **4** in $CDCl_3$

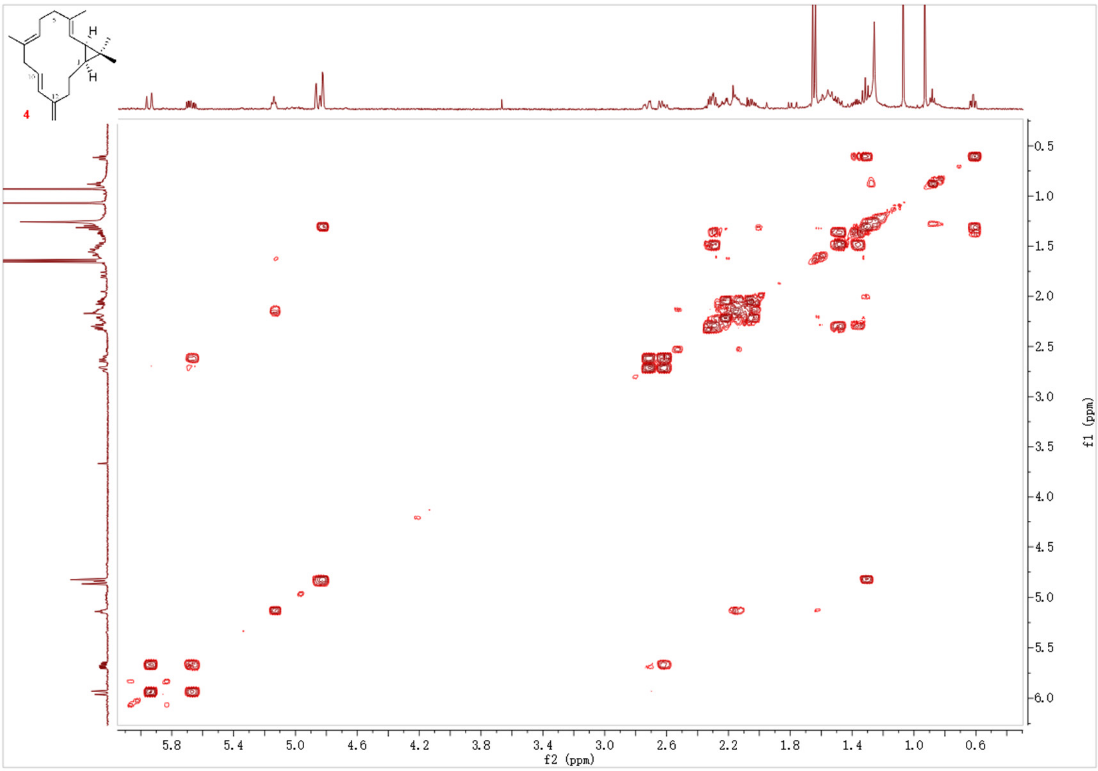

Figure S4d. ^{1}H-^{1}H COSY spectrum (600 MHz) of **4** in $CDCl_3$

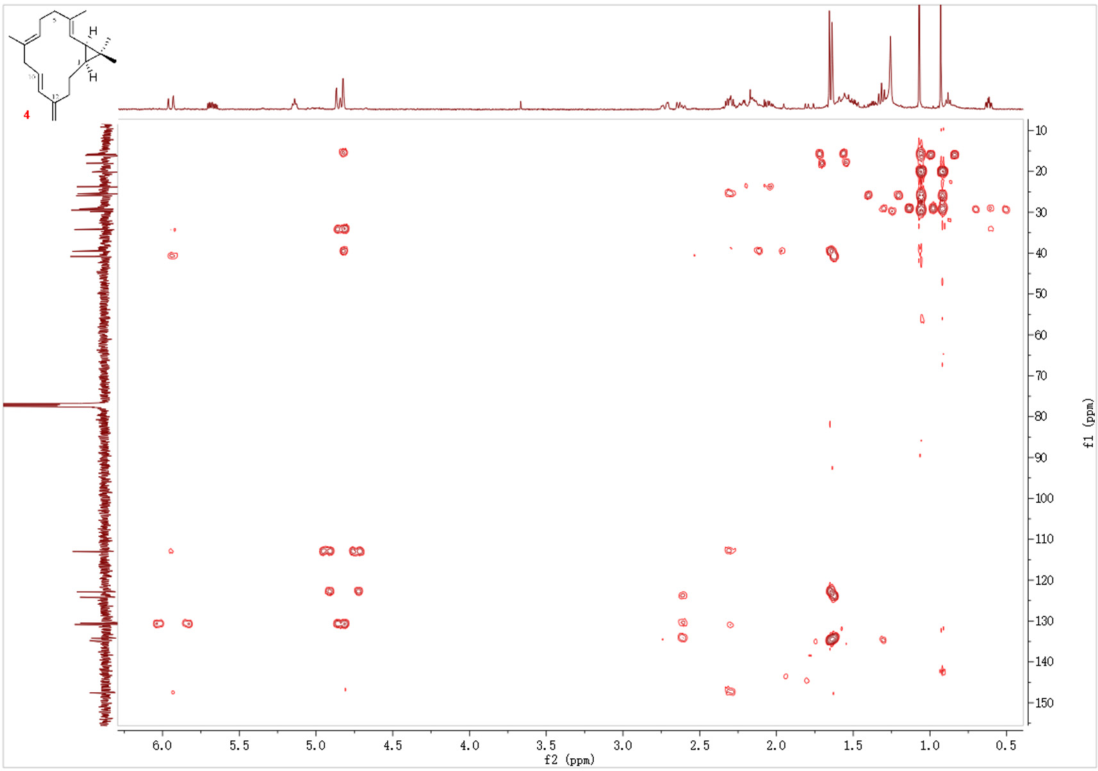

Figure S4e. HMBC spectrum (600 MHz) of **4** in $CDCl_3$

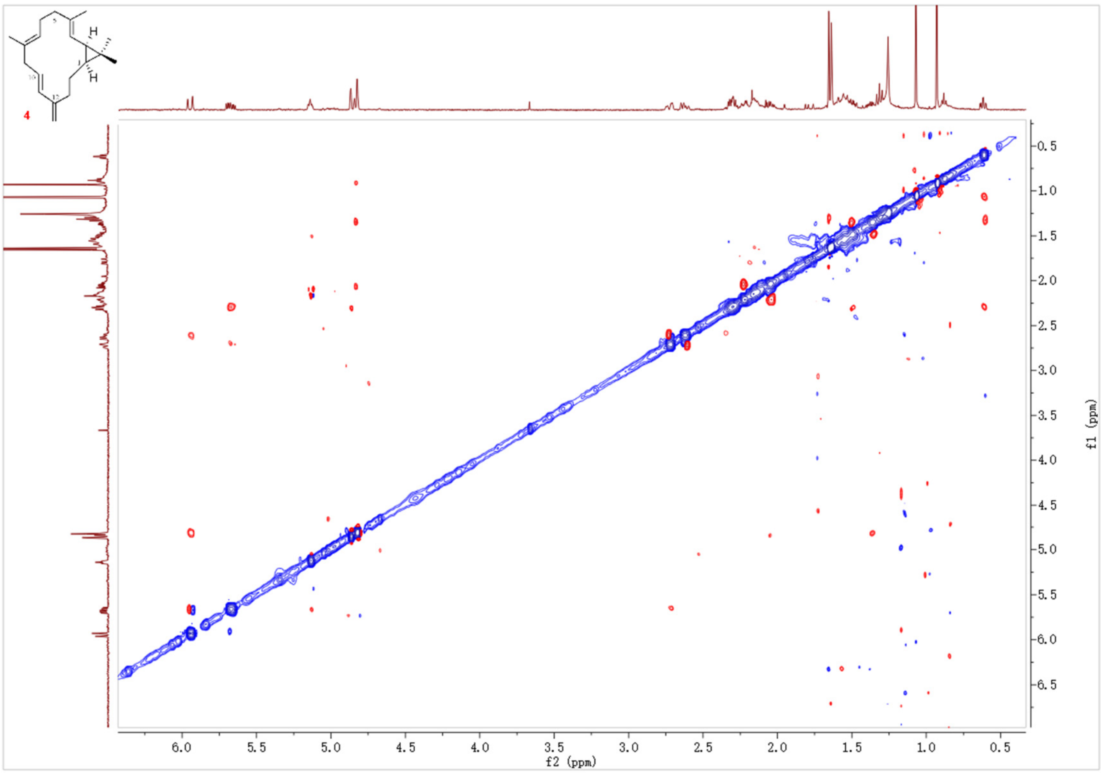

Figure S4f. NOESY spectrum (600 MHz) of **4** in $CDCl_3$

EI202200083_A8-1912-1-41 -c1#5 RT: 0.81
T: + c EI Full ms [49.50-800.50]
m/z= 48-803

m/z	Intensity	Relative	Theo. Mass	Delta (mmu)	RDB equiv.	Composition
131.0847	3164178.0	56.46	131.0855	-0.81	5.5	$C_{10}H_{11}$
227.1798	1609077.0	28.71	227.1794	0.39	6.5	$C_{17}H_{23}$
228.1848	429153.0	7.66	228.1873	-2.47	6.0	$C_{17}H_{24}$
255.2108	902465.0	16.10	255.2107	0.04	6.5	$C_{19}H_{27}$
270.2342	609755.0	10.88	270.2342	-0.01	6.0	$C_{20}H_{30}$

Figure S4g. HR-EIMS of **4**

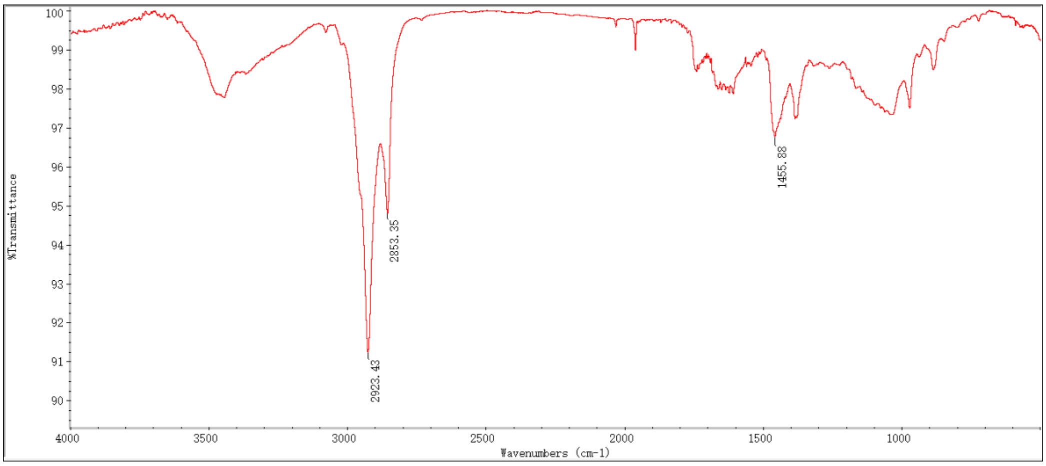

Figure S4h. IR spectrum of **4**

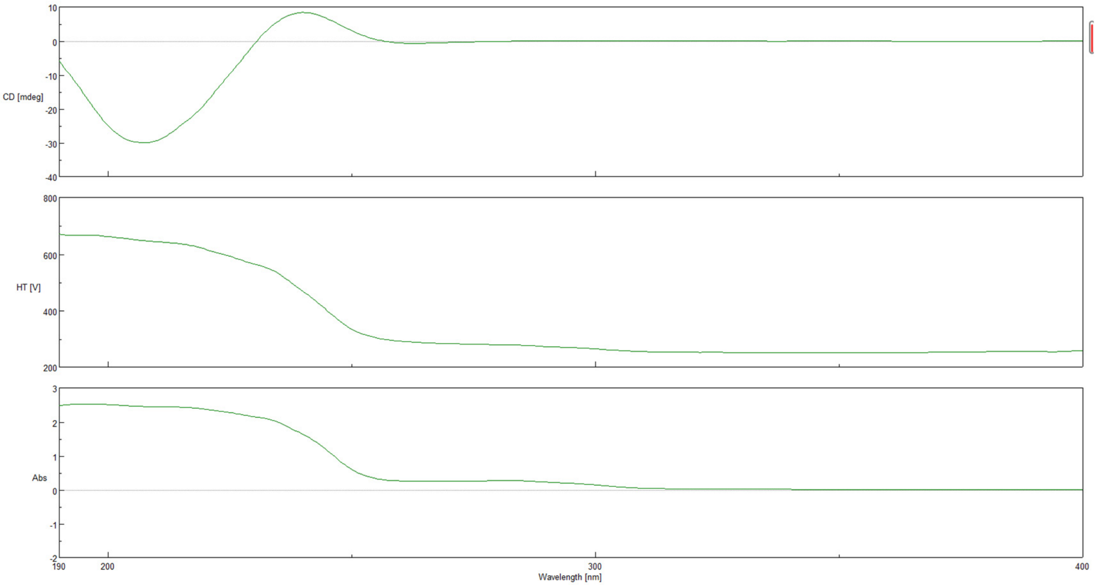

Figure S4i. ECD and UV spectra of **4**

5. QM-NMR calculation and DP4+ analysis of compound 3

Figure S5a. Structures of studied isomers for compound **3**

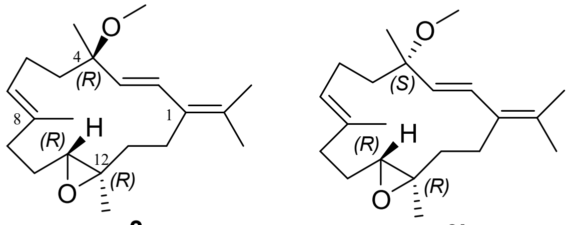

3a **3b**

Figure S5b. DP4+ results obtained using experimental data of **3** *versus* isomers 1 (**3a**) and 2 (**3b**).

Functional		Solvent?		Basis Set		Type of Data	
mPW1PW91		PCM		6-31G(d)		Shielding Tensors	
		DP4+	0.00%	100.00%	-	-	-
Nuclei	sp2?	xperimenta	Isomer 1	Isomer 2	Isomer 3	Isomer 4	Isomer 5
C		26.4	168.4	168.8			
C		61.6	69.4	67.8			
C		36.9	66.0	64.8			
C	x	132.5	157.0	156.8			
C	x	128.6	168.1	167.2			
C	x	129.5	129.9	131.6			
C	x	127.4	149.5	149.4			
C	x	130.5	116.7	116.7			
C		77.3	67.8	63.9			
C		41.7	134.1	134.0			
C		23	154.5	154.3			
C		24.4	67.62	69.03			
C		62.6	166.23	165.76			
C		37.6	65.16	65.31			
C	x	131.7	64.00	63.85			
C		21.5	171.69	171.62			
C		20.4	172.39	172.92			
C		23.3	169.51	169.58			
C		14.8	176.33	177.11			
C		16.3	175.41	175.92			
C		50.3	144.72	144.37			
H		2.295	29.95	29.85			
H		5.34	26.69	26.66			
H		2.215	30.02	29.99			
H		1.75	30.48111	30.47238			
H		2.79	29.58054	29.48324			
H		1.76	30.30201	30.29185			
H		5.71	26.75766	26.3238			
H		1.515	30.47556	30.48565			
H		6.48	25.42306	25.56866			
H		2.25	29.83487	29.82864			
H		1.81	30.43284	30.42748			
H		1.81	30.4112867	30.4589133			
H		1.31	30.96662	30.9350467			
H		1.7	30.62096	30.5196533			
H		1.3	30.9431533	30.91286			
H		3.07	28.9760133	28.9477067			

	A	B	C	D	E
4			Isomer 1	Isomer 2	Isomer 3
5	sDP4+ (H data)		0.06%	99.94%	-
6	sDP4+ (C data)		34.50%	65.50%	-
7	sDP4+ (all data)		0.03%	99.97%	-
8	uDP4+ (H data)		0.17%	99.83%	-
9	uDP4+ (C data)		35.79%	64.21%	-
10	uDP4+ (all data)		0.09%	99.91%	-
11	DP4+ (H data)		0.00%	100.00%	-
12	DP4+ (C data)		22.70%	77.30%	-
13	DP4+ (all data)		0.00%	100.00%	-

6. TDDFT-EDC calculations of compounds 1-4

Torsional sampling (MCMM) conformational searches using MMFFs force field were carried out by means of the conformational search module in the Macromodel applying an energy window of 21 kJ/mol, which afforded 145, 171, 125, 126 conformers for (12*S*)-**1**, (11*R*, 12*R*)-**2**, (4*S*, 11*R*, 12*R*)-**3**, (1*S*, 2*R*)-**4**, respectively. The Boltzmann populations of the conformers were obtained based on the potential energy provided by the MMFFs force field, which afforded 5, 5, 5, 4 conformers for re-optimization. The re-optimization and the following TDDFT calculations of the re-optimized geometries were all performed with Gaussian 09 at the B3LYP/6-311G(d,p) level with IEFPCM solvent model for acetonitrile. Frequency analysis was performed as well to confirm that the re-optimized geometries were at the energy minima. Finally, the SpecDis 1.62 software was used to obtain the Boltzmann-averaged ECD spectra and visualize the results.

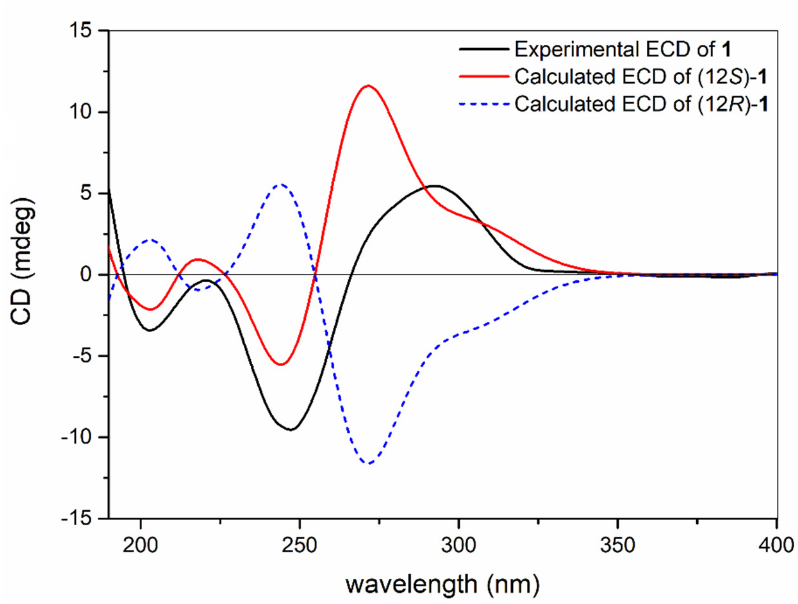

Figure S6a. Experimental ECD curve of **1**, and calculated ECD spectrum of (12*S*)-**1**

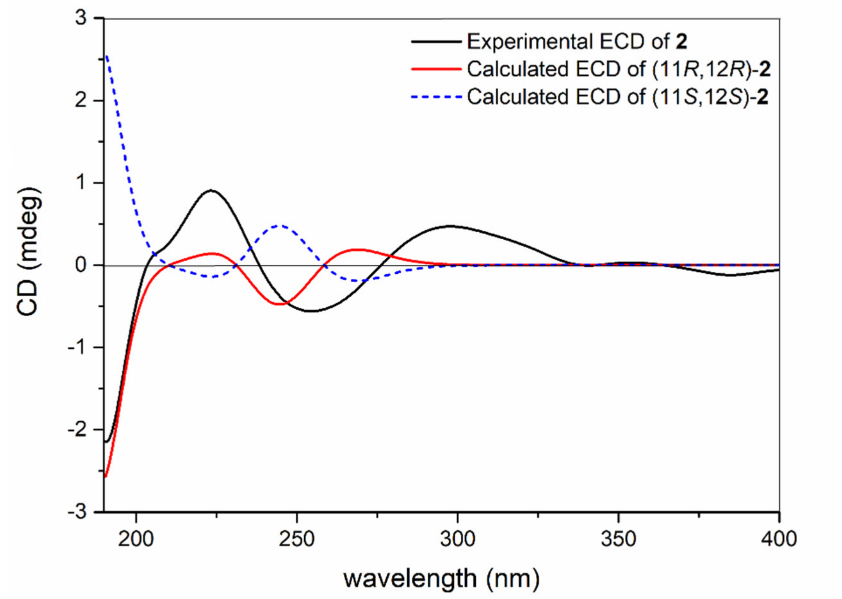

Figure S6b. Experimental ECD curve of **2**, and calculated ECD spectrum of (11*R*, 12*R*)-**2**

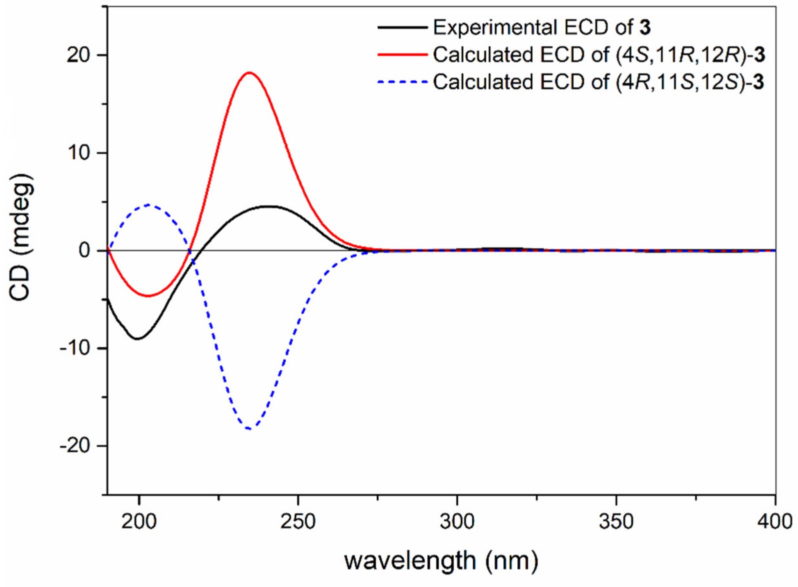

Figure S6c. Experimental ECD curve of **3**, and calculated ECD spectrum of (4*S*, 11*R*, 12*R*)-**3**

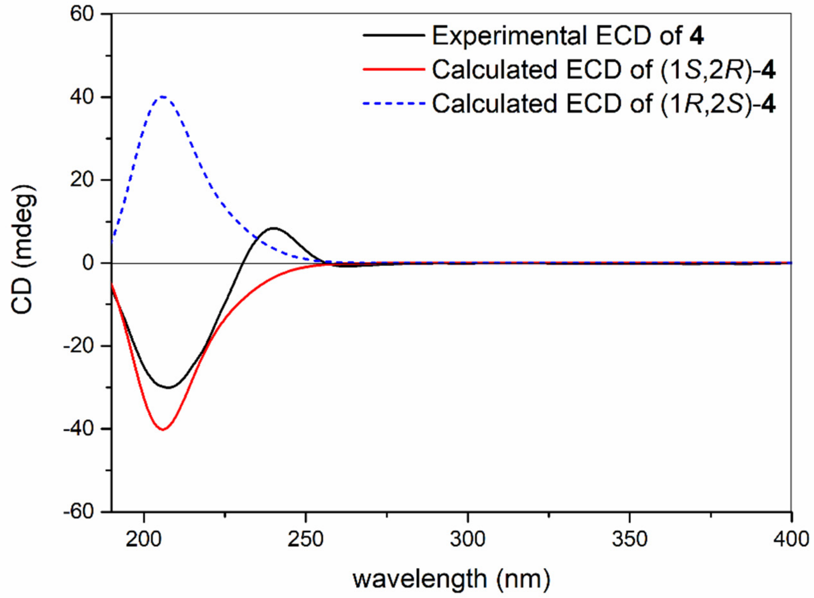

Figure S6d. Experimental ECD curve of **4**, and calculated ECD spectrum of (1*S*, 2*R*)-**4**

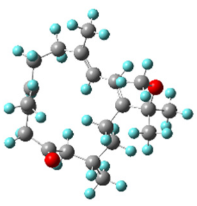

Conf. 1
3.16%
-971.22082194 a. u.

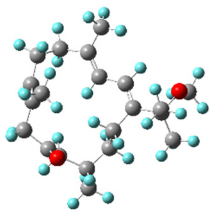

Conf. 2
9.43%
-971.22210426 a. u.

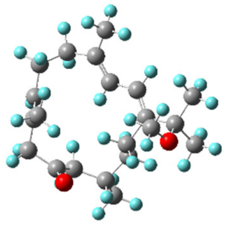

Conf. 3
16.36%
-971.22196749 a. u.

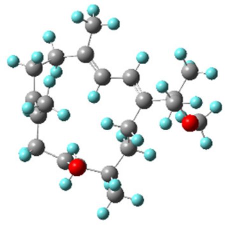

Conf. 4
5.73%
-971.22087028 a. u.

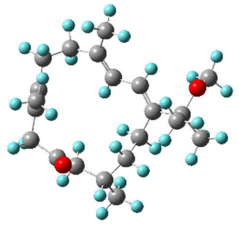

Conf. 5
65.32%
-971.22319110 a. u.

Conf. 1	Coordinates (Angstroms)		
atom	X	Y	Z
C	2.93858	-2.73139	-0.37242
C	3.25353	-1.26776	-0.22732
C	3.14115	-0.4776	0.84943
C	3.57909	0.9813	0.76691
C	2.38076	1.93369	0.76491
C	1.9124	2.41783	-0.59845
C	1.8815	-3.02857	-1.47663
C	0.44522	-2.83282	-1.02916
C	-0.08728	-1.59638	-0.9677
C	0.53746	3.11018	-0.66008
C	-0.6164	2.11737	-0.91461
C	-1.44456	-1.23358	-0.56766
C	-0.80447	1.05656	0.188
C	-1.82645	-0.02793	-0.0984
C	-3.32664	0.24448	0.14523
C	-3.58568	1.30422	1.22865
C	-3.99279	0.68716	-1.16861
O	-4.02832	-0.9794	0.46707
C	-0.28146	-4.08826	-0.62352
C	2.63491	-0.91462	2.20027
C	0.53523	4.20279	-1.74009
O	1.86503	2.29552	1.80501
C	-3.76931	-1.55618	1.74101
H	3.86512	-3.24526	-0.65875
H	2.61891	-3.17631	0.5725
H	3.65134	-0.81449	-1.13526
H	4.17466	1.13923	-0.13417
H	4.18944	1.23212	1.64034
H	2.69785	3.11945	-0.91531
H	1.9787	1.58959	-1.31344
H	2.0145	-4.06267	-1.8082
H	2.09824	-2.39411	-2.34217
H	0.5649	-0.77814	-1.25737
H	0.37077	3.58748	0.31183
H	-1.54122	2.69217	-1.03118
H	-0.44509	1.61519	-1.87539
H	-2.21447	-1.99155	-0.67038
H	0.15154	0.56662	0.37319
H	-1.05408	1.56689	1.12175
H	-4.66074	1.36757	1.41372
H	-3.08638	1.0717	2.17129
H	-3.24208	2.28757	0.9024
H	-5.05367	0.89056	-0.99778
H	-3.52393	1.58907	-1.56597
H	-3.90268	-0.10587	-1.91342
H	0.28083	-4.61961	0.15379
H	-1.2829	-3.89821	-0.23846
H	-0.36192	-4.77913	-1.47108
H	1.87562	-0.22095	2.5722
H	2.20696	-1.91638	2.18631
H	3.45079	-0.90264	2.9329
H	-0.43122	4.71317	-1.77921
H	1.30471	4.95517	-1.54432
H	0.72766	3.77765	-2.73114
H	-4.25307	-2.5342	1.73892
H	-2.69728	-1.69317	1.9203
H	-4.19416	-0.96043	2.55637

Conf. 2	Coordinates (Angstroms)		
atom	X	Y	Z
C	2.9733	-2.70612	-0.36256
C	3.29655	-1.2395	-0.28175
C	3.22462	-0.4093	0.76798
C	3.65123	1.04698	0.61315
C	2.4441	1.98755	0.58686
C	1.90068	2.3344	-0.79
C	1.84028	-3.03053	-1.38192
C	0.43967	-2.85282	-0.82787
C	-0.11571	-1.62662	-0.75513
C	0.50347	2.9805	-0.85838
C	-0.62972	1.93583	-0.94584
C	-1.43686	-1.28379	-0.23902
C	-0.80939	1.07204	0.31944
C	-1.80945	-0.0659	0.20281
C	-3.23181	0.15494	0.74878
C	-3.21286	0.0131	2.28075

C	-3.8075	1.53181	0.36974
O	-4.12526	-0.89792	0.327
C	-0.21926	-4.11376	-0.33234
C	2.77051	-0.79453	2.15276
C	0.42187	3.94207	-2.05422
O	1.97973	2.4442	1.61372
C	-4.57754	-0.87058	-1.02238
H	3.87795	-3.22881	-0.6979
H	2.72069	-3.12313	0.61485
H	3.65752	-0.82088	-1.22118
H	4.22735	1.16816	-0.30633
H	4.27519	1.34252	1.46214
H	2.64986	3.02708	-1.20087
H	1.9626	1.44861	-1.43204
H	1.9636	-4.06596	-1.71326
H	1.98054	-2.40157	-2.26693
H	0.49093	-0.80275	-1.11737
H	0.36603	3.56221	0.05989
H	-1.56627	2.45997	-1.15779
H	-0.44302	1.28629	-1.80979
H	-2.18197	-2.07107	-0.20666
H	0.15422	0.64236	0.59753
H	-1.08567	1.72889	1.14945
H	-4.20806	0.21359	2.68652
H	-2.92059	-1.0031	2.55228
H	-2.5062	0.71102	2.73446
H	-4.83651	1.60293	0.73163
H	-3.23301	2.3352	0.83316
H	-3.80812	1.69881	-0.70839
H	0.41954	-4.61183	0.40699
H	-1.18977	-3.93921	0.13121
H	-0.35278	-4.82774	-1.15371
H	2.0174	-0.09311	2.52358
H	2.35131	-1.7992	2.19485
H	3.61126	-0.74572	2.85501
H	-0.55499	4.43163	-2.09783
H	1.18614	4.72175	-1.98804
H	0.56965	3.40862	-2.99953
H	-5.08602	-1.82085	-1.19264
H	-5.29086	-0.05851	-1.19941
H	-3.75082	-0.78109	-1.73493

Conf. 3	Coordinates (Angstroms)		
atom	X	Y	Z
C	3.16654	-2.43402	-0.2557
C	3.34404	-0.95519	-0.04902
C	3.05602	-0.20425	1.02276
C	3.35611	1.29114	1.00201
C	2.07962	2.11247	0.80254
C	1.72752	2.45336	-0.63633
C	2.22153	-2.77759	-1.44618
C	0.74857	-2.76599	-1.08611
C	0.06811	-1.60322	-1.02918
C	0.30441	2.97341	-0.9159
C	-0.70134	1.83657	-1.19686
C	-1.33784	-1.42168	-0.67965
C	-1.03617	0.95897	0.02831
C	-1.88915	-0.27067	-0.24156
C	-3.38149	-0.15829	0.10455
C	-4.05213	0.95376	-0.71656
C	-4.17702	-1.45761	-0.08374
O	-3.52424	0.31299	1.47369
C	0.16146	-4.1138	-0.75732
C	2.45358	-0.71245	2.30759
C	0.32502	3.95873	-2.09525
O	1.41677	2.48506	1.75128
C	-3.03095	-0.53928	2.49875
H	4.15153	-2.86059	-0.48293
H	2.81453	-2.93457	0.64891
H	3.78987	-0.44678	-0.90386
H	4.06285	1.51628	0.20065
H	3.79506	1.59671	1.95626
H	2.46415	3.22269	-0.91103
H	1.97122	1.59749	-1.27547
H	2.48838	-3.77031	-1.8207
H	2.41863	-2.07346	-2.2607
H	0.63188	-0.70432	-1.25454
H	-0.02796	3.51427	-0.0229
H	-1.62408	2.28181	-1.5798
H	-0.30912	1.21276	-2.00908
H	-1.97325	-2.29309	-0.78542
H	-0.1062	0.62627	0.49248
H	-1.53816	1.57846	0.77529
H	-5.1061	1.02758	-0.437
H	-3.58362	1.91923	-0.52275
H	-3.98643	0.73879	-1.78521
H	-5.20001	-1.29532	0.26342
H	-4.21674	-1.74155	-1.13742
H	-3.75681	-2.29485	0.47521
H	0.7559	-4.60694	0.02096
H	-0.8689	-4.06473	-0.40649
H	0.19592	-4.77256	-1.63315
H	1.58814	-0.10826	2.59587
H	2.13758	-1.75311	2.24349
H	3.17805	-0.62592	3.12607
H	-0.67298	4.36328	-2.28554
H	0.99653	4.79944	-1.89796
H	0.66586	3.4676	-3.01332
H	-3.62385	-1.45583	2.59746
H	-1.98265	-0.81539	2.33693
H	-3.10785	0.02436	3.42963

Conf. 4	Coordinates (Angstroms)		
atom	X	Y	Z
C	3.47477	-2.11049	-0.46038
C	3.49018	-0.61412	-0.31235
C	3.26941	0.13486	0.77671
C	3.36873	1.65422	0.68399
C	1.98235	2.30398	0.67569
C	1.38012	2.56673	-0.69507
C	2.42439	-2.61109	-1.49643
C	1.02389	-2.75527	-0.93364
C	0.21751	-1.68099	-0.82063
C	-0.11874	2.91784	-0.75531
C	-1.02284	1.67284	-0.87844
C	-1.14637	-1.65426	-0.29753
C	-1.0272	0.75375	0.35925
C	-1.7667	-0.56763	0.20775
C	-3.2007	-0.63171	0.75498
C	-3.93984	-1.9392	0.43362
C	-3.20716	-0.43442	2.2806
O	-3.95172	0.50955	0.25928
C	0.65761	-4.1443	-0.47979
C	2.93401	-0.39619	2.14677
C	-0.39116	3.87626	-1.92532
O	1.4257	2.61002	1.71248
C	-4.35431	0.45809	-1.10373
H	4.46448	-2.42263	-0.81667
H	3.31753	-2.61418	0.49593
H	3.73485	-0.08725	-1.23462

H	3.90943	1.93623	-0.22187
H	3.90694	2.04611	1.55214
H	1.97371	3.40571	-1.08695
H	1.62135	1.72676	-1.3562
H	2.75525	-3.58097	-1.87988
H	2.4219	-1.92325	-2.34796
H	0.62803	-0.73243	-1.15069
H	-0.37051	3.43609	0.17678
H	-2.04571	2.00959	-1.06351
H	-0.71876	1.10487	-1.76656
H	-1.68562	-2.59401	-0.32421
H	0.00259	0.52933	0.64234
H	-1.45036	1.32104	1.19049
H	-4.98062	-1.84457	0.75229
H	-3.92514	-2.18027	-0.63029
H	-3.49861	-2.77708	0.97647
H	-4.23542	-0.47362	2.64838
H	-2.62792	-1.21985	2.77079
H	-2.78659	0.53265	2.55785
H	1.40578	-4.52148	0.22752
H	-0.31566	-4.19799	0.00716
H	0.65826	-4.83965	-1.3275
H	2.05682	0.11424	2.55496
H	2.73829	-1.46785	2.14793
H	3.7588	-0.19929	2.84195
H	-1.44755	4.15615	-1.96428
H	0.19662	4.79409	-1.83172
H	-0.13444	3.4114	-2.88354
H	-4.70644	1.45836	-1.36062
H	-3.52614	0.19422	-1.77119
H	-5.17466	-0.25051	-1.26326

Conf. 5	Coordinates (Angstroms)		
atom	X	Y	Z
C	3.17426	-2.59145	-0.55996
C	3.37979	-1.10309	-0.60385
C	3.51721	-0.24099	0.41252
C	3.75128	1.23755	0.12176
C	2.50247	2.06716	0.42721
C	1.63578	2.45057	-0.76088
C	1.88641	-3.05027	-1.30183
C	0.60409	-2.80354	-0.53682
C	-0.11565	-1.68351	-0.75706
C	0.29276	3.10836	-0.40998
C	-0.7214	2.10725	0.19008
C	-1.37411	-1.31105	-0.1279
C	-1.34764	1.10683	-0.81473
C	-1.98149	-0.10555	-0.16211
C	-3.33779	0.05266	0.56438
C	-3.11301	0.33616	2.05924
C	-4.21702	1.15955	-0.04123
O	-4.06193	-1.20073	0.58185
C	0.24228	-3.86746	0.4673
C	3.48177	-0.60469	1.87464
C	-0.27523	3.88038	-1.60958
O	2.236	2.40058	1.56643
C	-4.59188	-1.66045	-0.65558
H	4.02689	-3.06946	-1.05919
H	3.1674	-2.97013	0.46417
H	3.44099	-0.6956	-1.6128
H	4.03952	1.37158	-0.92239
H	4.55628	1.61742	0.7593
H	2.24881	3.13881	-1.36021
H	1.51351	1.56697	-1.39735
H	1.97831	-4.12247	-1.50803
H	1.84794	-2.54492	-2.27202
H	0.28437	-0.99738	-1.49778
H	0.50459	3.83358	0.38272
H	-0.22503	1.55076	0.98921
H	-1.51868	2.68427	0.66999
H	-1.89878	-2.09494	0.40839
H	-2.08367	1.6306	-1.43012
H	-0.57279	0.7691	-1.50466
H	-4.07708	0.44231	2.56432
H	-2.56815	-0.49303	2.51464
H	-2.53776	1.25054	2.20838
H	-5.19782	1.13694	0.43999
H	-3.78025	2.14437	0.13168
H	-4.35782	1.04046	-1.1172
H	1.07748	-4.05223	1.15257
H	-0.62875	-3.61351	1.07022
H	0.04343	-4.82012	-0.03828
H	4.468	-0.44714	2.32761
H	2.78616	0.0425	2.41564
H	3.19307	-1.64102	2.04652
H	-1.2321	4.34665	-1.35627
H	0.41068	4.67331	-1.9215
H	-0.44032	3.22884	-2.47279
H	-4.96367	-2.66983	-0.47304
H	-5.42611	-1.04058	-1.00188
H	-3.82835	-1.70216	-1.43996

Figure S6e. Re-optimized conformers of (12*S*)-**1** calculated at the B3LYP/6-311G(d,p) level with IEFPCM solvent model for acetonitrile.

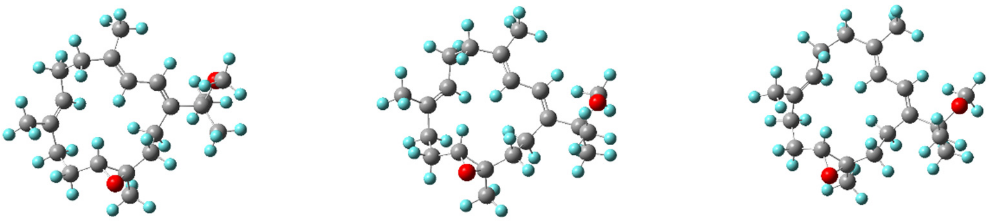

Conf. 1 20.79% -971.16409466 a. u.

Conf. 2 4.59% -971.16296862 a. u.

Conf. 3 1.77% -971.15570297 a. u.

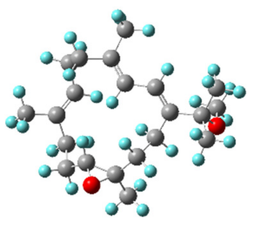

Conf. 4
70.48%
-971.16454601 a. u.

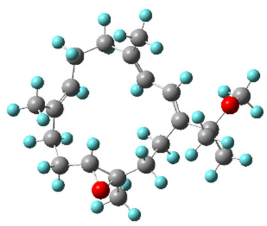

Conf. 5
2.38%
-971.15664626 a. u.

Conf. 1	Coordinates (Angstroms)		
atom	X	Y	Z
C	2.66625	2.8229	0.48781
C	2.75898	1.37958	0.91351
C	3.6683	0.46786	0.53723
C	3.58609	-0.95919	1.05036
C	3.33129	-2.03874	-0.03037
C	1.99554	-1.86952	-0.71332
C	1.6832	3.0661	-0.6958
C	0.21718	2.86385	-0.35861
C	-0.31485	1.62531	-0.37695
C	0.75107	-2.60014	-0.39352
C	-0.56942	-1.90152	-0.68544
C	-1.69395	1.23563	-0.10426
C	-1.07988	-1.10023	0.5387
C	-2.09184	-0.00491	0.24365
C	-3.58057	-0.30575	0.48778
C	-3.99074	-1.697	-0.03027
C	-3.88335	-0.20656	1.99256
O	-4.42033	0.71554	-0.09108
C	-0.54261	4.10767	0.01976
C	4.82469	0.7545	-0.38989
C	0.70001	-3.69314	0.65091
O	1.52523	-2.93211	-1.5752
C	-4.58758	0.69933	-1.50497
H	3.64636	3.20202	0.18754
H	2.34791	3.43169	1.34166
H	1.96953	1.04265	1.58143
H	4.5312	-1.2109	1.54774
H	2.8023	-1.02726	1.81069
H	3.40291	-3.02598	0.43023
H	4.1161	-1.99798	-0.79359
H	1.86078	-0.88681	-1.16359
H	1.97244	2.40272	-1.5162
H	1.83016	4.09148	-1.05143
H	0.36625	0.82032	-0.63211
H	-0.42002	-1.23168	-1.53556
H	-1.31324	-2.64355	-0.99372
H	-2.46224	1.99458	-0.20508
H	-0.21529	-0.64032	1.02577
H	-1.49242	-1.79736	1.27212
H	-5.06983	-1.82114	0.09144
H	-3.74259	-1.83621	-1.08373
H	-3.49783	-2.48708	0.53799
H	-4.92825	-0.46698	2.18087
H	-3.24823	-0.88093	2.57054
H	-3.70827	0.81492	2.33585
H	-0.57058	4.81355	-0.81884
H	-1.56679	3.90503	0.33239
H	-0.038	4.62775	0.84272
H	5.76667	0.41489	0.0556
H	4.71689	0.21455	-1.33699
H	4.92478	1.81303	-0.62829
H	-0.04222	-4.44325	0.36064
H	1.66142	-4.19439	0.75609
H	0.40906	-3.2946	1.6263
H	-5.22103	-0.13186	-1.83289
H	-5.08359	1.63593	-1.76417
H	-3.6304	0.64947	-2.03444

Conf. 2	Coordinates (Angstroms)		
atom	X	Y	Z
C	2.52434	2.92305	0.44295
C	2.63295	1.50382	0.9411
C	3.58407	0.60271	0.65242
C	3.52069	-0.7983	1.23504
C	3.3864	-1.94384	0.20205
C	2.10019	-1.8708	-0.58556
C	1.62128	3.08146	-0.81635
C	0.14402	2.8296	-0.57734
C	-0.33625	1.57043	-0.59943
C	0.86605	-2.63782	-0.31583
C	-0.45437	-2.01082	-0.73972
C	-1.7175	1.13055	-0.42628
C	-1.05354	-1.13008	0.38552
C	-2.09218	-0.10819	-0.04492
C	-3.58608	-0.49323	-0.06211
C	-3.9578	-1.03797	-1.45157
C	-3.96242	-1.53223	1.00815
O	-4.42123	0.68	0.06606
C	-0.6897	4.04927	-0.28752
C	4.77582	0.88114	-0.23147
C	0.78185	-3.6712	0.78537
O	1.74009	-2.99896	-1.41676
C	-4.47148	1.29943	1.34578
H	3.51089	3.32245	0.19388
H	2.12815	3.55743	1.24387
H	1.82147	1.17555	1.58663
H	4.43859	-0.98299	1.80735
H	2.69006	-0.85883	1.9447
H	3.46119	-2.90078	0.72232
H	4.22375	-1.9115	-0.50365
H	1.9595	-0.92078	-1.09995
H	1.99466	2.40071	-1.58682
H	1.75373	4.09844	-1.20077
H	0.39283	0.78793	-0.7836
H	-0.27412	-1.40548	-1.63156
H	-1.15687	-2.80073	-1.0278
H	-2.50466	1.84307	-0.64958
H	-0.22989	-0.59027	0.86007
H	-1.46502	-1.77731	1.16138

H	-5.01004	-1.3353	-1.46653
H	-3.79963	-0.26443	-2.20542
H	-3.34701	-1.90465	-1.71174
H	-5.04874	-1.64934	1.02171
H	-3.52477	-2.50512	0.77809
H	-3.63268	-1.24342	2.00812
H	-0.68422	4.73425	-1.14369
H	-1.72501	3.81061	-0.04452
H	-0.26732	4.60794	0.5561
H	5.70296	0.57746	0.26772
H	4.72233	0.30671	-1.1629
H	4.86559	1.93277	-0.50258
H	0.0808	-4.46027	0.49631
H	1.74945	-4.13377	0.97599
H	0.42101	-3.22766	1.71703
H	-3.47189	1.51527	1.73842
H	-5.00666	2.24049	1.21006
H	-5.01744	0.69135	2.07533

Conf. 3	Coordinates (Angstroms)		
atom	X	Y	Z
C	-2.32811	2.91043	0.33956
C	-2.66468	1.68257	-0.48236
C	-3.60816	0.77504	-0.19737
C	-3.78586	-0.46169	-1.05609
C	-3.60103	-1.79637	-0.2927
C	-2.27775	-1.88004	0.43015
C	-1.1136	3.71846	-0.14388
C	0.27165	3.10883	-0.02445
C	0.47974	1.79188	0.17428
C	-1.06849	-2.6031	-0.01355
C	0.27346	-2.08493	0.48321
C	1.76983	1.11302	0.29006
C	0.92159	-1.09784	-0.5174
C	2.00353	-0.1919	0.03966
C	3.40392	-0.77688	0.32713
C	3.48456	-1.22972	1.79479
C	3.76787	-1.95498	-0.59185
O	4.4193	0.24981	0.24669
C	1.38256	4.1132	-0.20282
C	-4.53472	0.89971	0.9885
C	-1.03717	-3.42766	-1.28078
O	-1.91308	-3.14179	1.03648
C	4.74132	0.73925	-1.04968
H	-2.16891	2.62654	1.3867
H	-3.18682	3.59327	0.34857
H	-2.06116	1.52236	-1.37344
H	-4.79473	-0.4682	-1.48795
H	-3.08213	-0.42389	-1.89296
H	-3.71555	-2.62802	-0.99069
H	-4.39699	-1.90831	0.45228
H	-2.09343	-1.0363	1.09413
H	-1.10372	4.66885	0.40432
H	-1.26703	4.00671	-1.19486
H	-0.39723	1.1608	0.2546
H	0.11019	-1.58705	1.44277
H	0.93613	-2.93624	0.67429
H	2.61898	1.70629	0.61265
H	0.13025	-0.47184	-0.93415
H	1.32394	-1.66253	-1.36305
H	4.4722	-1.65215	1.99954
H	3.32485	-0.374	2.45351
H	2.72901	-1.98434	2.01943
H	4.80393	-2.24622	-0.40286
H	3.13431	-2.8198	-0.38765

H	3.66654	-1.70808	-1.65046
H	1.34741	4.87184	0.58779
H	2.37379	3.66187	-0.20076
H	1.26093	4.64985	-1.15111
H	-4.2847	0.18116	1.77782
H	-4.50888	1.89533	1.4318
H	-5.56743	0.68727	0.69073
H	-0.34486	-4.26647	-1.15838
H	-2.02034	-3.83368	-1.5164
H	-0.69578	-2.83252	-2.13164
H	5.28717	-0.00115	-1.64444
H	3.85051	1.05504	-1.60337
H	5.38687	1.60579	-0.89863

Conf. 4	Coordinates (Angstroms)		
atom	X	Y	Z
C	2.71426	2.74152	0.50437
C	2.68641	1.31639	0.99462
C	3.57826	0.34549	0.7466
C	3.36337	-1.05446	1.29458
C	3.17382	-2.1575	0.22466
C	1.95419	-1.9228	-0.63418
C	1.8808	2.97252	-0.79195
C	0.37907	2.85996	-0.60925
C	-0.21875	1.65283	-0.66844
C	0.63643	-2.57518	-0.48475
C	-0.5888	-1.80733	-0.95941
C	-1.64073	1.35406	-0.53095
C	-1.24631	-1.00804	0.19443
C	-2.15078	0.14849	-0.20378
C	-3.66196	-0.1126	-0.13094
C	-4.53165	1.12725	-0.37995
C	-4.06448	-1.21203	-1.12621
O	-3.99354	-0.69555	1.1594
C	-0.3388	4.15334	-0.32821
C	4.83735	0.53673	-0.06363
C	0.38211	-3.64934	0.54967
O	1.54433	-2.96665	-1.54761
C	-3.76399	0.11613	2.3038
H	3.73897	3.06216	0.3004
H	2.33501	3.4033	1.29118
H	1.81562	1.04712	1.58812
H	4.23113	-1.33514	1.90463
H	2.49586	-1.05348	1.96132
H	3.11494	-3.12838	0.72058
H	4.05156	-2.19876	-0.4297
H	1.93976	-0.9393	-1.10181
H	2.21889	2.25435	-1.54439
H	2.12057	3.97043	-1.17432
H	0.43626	0.80513	-0.83718
H	-0.28064	-1.13508	-1.76362
H	-1.30682	-2.50998	-1.39483
H	-2.32474	2.17231	-0.72465
H	-0.44662	-0.60915	0.82624
H	-1.81867	-1.69076	0.82473
H	-5.57833	0.85874	-0.21981
H	-4.28472	1.95413	0.28753
H	-4.42508	1.47972	-1.40784
H	-5.13673	-1.40658	-1.043
H	-3.84115	-0.90547	-2.1502
H	-3.53531	-2.14224	-0.9155
H	-0.24271	4.84001	-1.17749
H	-1.39876	4.02067	-0.11244
H	0.11586	4.66107	0.53061
H	4.80001	-0.03598	-0.99685
H	5.01606	1.57848	-0.32838

H	5.70935	0.17101	0.49048
H	-0.37232	-4.34889	0.17632
H	1.28684	-4.21492	0.76915
H	0.00726	-3.21972	1.48216
H	-3.9501	-0.51777	3.17189
H	-2.73222	0.48402	2.34796
H	-4.4444	0.97406	2.34777

Conf. 5	Coordinates (Angstroms)		
atom	X	Y	Z
C	-2.74365	2.89377	-0.24394
C	-2.9617	1.47411	-0.70675
C	-3.65815	0.49382	-0.11673
C	-3.79944	-0.85324	-0.80413
C	-3.37848	-2.0826	0.03078
C	-1.96733	-2.00523	0.56033
C	-1.40857	3.49487	-0.75368
C	-0.16368	2.96332	-0.06947
C	0.4542	1.85996	-0.53543
C	-0.76171	-2.64011	-0.01546
C	0.58461	-2.02798	0.33954
C	1.69175	1.26175	-0.03973
C	1.16222	-1.08015	-0.74601
C	2.08777	-0.01955	-0.18419
C	3.48928	-0.42841	0.32405
C	3.44845	-0.7637	1.82439
C	4.07555	-1.61926	-0.45346
O	4.39928	0.69547	0.27288
C	0.28243	3.72818	1.14927
C	-4.38929	0.64949	1.19422
C	-0.80357	-3.49263	-1.26266
O	-1.4236	-3.21204	1.14307
C	4.81602	1.136	-1.01413
H	-2.79282	2.97221	0.84574
H	-3.55519	3.52596	-0.63007
H	-2.51568	1.2447	-1.67381
H	-4.851	-0.99888	-1.08466
H	-3.22671	-0.83942	-1.73621
H	-3.50988	-2.98352	-0.57197
H	-4.04698	-2.19293	0.89232
H	-1.77713	-1.12272	1.17093
H	-1.44894	4.58104	-0.62035
H	-1.34371	3.31583	-1.83192
H	0.	1.38053	-1.39836
H	0.45596	-1.47578	1.27331
H	1.28695	-2.84227	0.54903
H	2.38997	1.93029	0.45486
H	0.32406	-0.5869	-1.24049
H	1.66604	-1.67226	-1.51481
H	4.45152	-1.0303	2.16833
H	3.10447	0.10521	2.3888
H	2.77743	-1.59829	2.02998
H	5.10691	-1.78187	-0.1312
H	3.51325	-2.53252	-0.25247
H	4.07266	-1.45499	-1.53268
H	-0.53943	3.80927	1.87046
H	1.12629	3.26458	1.65987
H	0.56044	4.75482	0.88258
H	-4.00877	-0.0414	1.95423
H	-4.31837	1.6595	1.59693
H	-5.45249	0.41384	1.06654
H	-0.05945	-4.29186	-1.18908
H	-1.78105	-3.95286	-1.40378
H	-0.57011	-2.89858	-2.15026
H	5.36799	2.06365	-0.85567
H	5.48169	0.41443	-1.50008
H	3.96633	1.33955	-1.6746

Figure S6f. Re-optimized conformers of (11*R*, 12*R*)-**2** calculated at the B3LYP/6-311G(d,p) level with IEFPCM solvent model for acetonitrile.

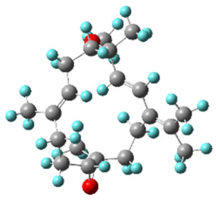

Conf. 1
0.02%
-971.15464835 a. u.

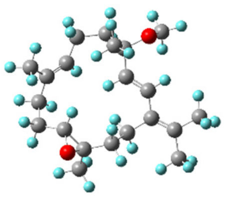

Conf. 2
74.32%
-971.15296407 a. u.

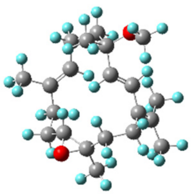

Conf. 3
0.00%
-971.14545343 a. u.

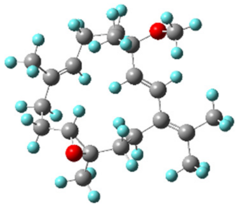

Conf. 4
25.66%
-971.15480698 a. u.

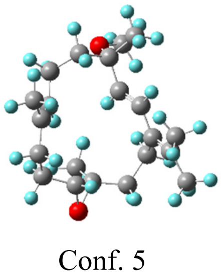

Conf. 5
0.01%
-971.15368466 a. u.

Conf. 1	Coordinates (Angstroms)		
atom	X	Y	Z
C	-2.26388	2.17362	-0.8949
C	-0.82079	2.05678	-1.32887
C	0.2131	2.82783	-0.95775
C	1.56693	2.67386	-1.62539
C	2.76526	2.36661	-0.70213
C	2.91193	0.91849	-0.27961
C	-3.09656	0.89481	-1.08702
C	-2.98224	-0.17854	0.02399
C	-1.54976	-0.63551	0.25064
C	2.5759	0.32229	1.03506
C	2.46611	-1.19503	1.16704
C	-0.97823	-1.73547	-0.26985
C	1.06434	-1.8439	1.29483
C	0.36293	-2.27754	0.00686
C	0.88969	-3.24783	-0.78752
C	2.1513	-4.01043	-0.45278
C	0.28153	-3.69845	-2.09766
C	-3.93687	-1.33503	-0.32156
C	0.09277	3.96307	0.03242
C	1.94927	1.13654	2.14858
O	3.93843	0.68426	0.70072
O	-3.43852	0.51303	1.20874
C	-3.53823	-0.25286	2.39481
H	-2.74152	2.96365	-1.4955
H	-2.35388	2.49543	0.14747
H	-0.62889	1.29273	-2.08402
H	1.50529	1.90434	-2.4062
H	1.79483	3.61653	-2.14674
H	2.75663	3.01536	0.1788
H	3.68968	2.61579	-1.24184
H	2.94598	0.21981	-1.12247
H	-2.84226	0.42538	-2.04625
H	-4.15697	1.17123	-1.13682
H	-0.98096	0.03102	0.8952
H	3.01702	-1.63806	0.33346
H	3.03831	-1.44614	2.07189
H	-1.58957	-2.34067	-0.9335
H	0.40578	-1.1855	1.86662
H	1.17717	-2.73608	1.92515
H	2.00687	-5.07794	-0.66883
H	2.99866	-3.68584	-1.07424
H	2.45563	-3.9216	0.59233
H	-0.15908	-4.7024	-2.00611
H	-0.48256	-3.02479	-2.48869
H	1.0652	-3.77798	-2.86332
H	-3.86082	-2.15699	0.3963
H	-4.96821	-0.96719	-0.32631
H	-3.71937	-1.73998	-1.31514
H	-0.91791	4.06422	0.43393
H	0.77552	3.8394	0.88252
H	0.35953	4.91822	-0.44253
H	2.22954	0.72293	3.12515
H	2.28874	2.17381	2.11775
H	0.8557	1.13181	2.07958
H	-2.61153	-0.80135	2.61736
H	-3.73137	0.4568	3.20446
H	-4.36738	-0.97416	2.35946

Conf. 2	Coordinates (Angstroms)		
atom	X	Y	Z
C	-1.59768	3.09968	-0.51245
C	-2.1291	1.75253	-0.93882
C	-3.15764	1.05795	-0.42315
C	-3.57027	-0.27027	-1.03604
C	-3.58005	-1.47059	-0.06025
C	-2.24029	-1.69934	0.60205
C	-0.06665	3.28432	-0.62148
C	0.85647	2.51831	0.37018
C	0.97175	1.04282	0.01942
C	-1.23242	-2.72017	0.23255
C	0.22225	-2.42095	0.56728
C	2.107	0.32603	0.10759
C	1.0296	-1.85771	-0.62668
C	2.27985	-1.10172	-0.20016
C	3.48676	-1.71815	-0.08253
C	3.69951	-3.19461	-0.32909
C	4.77426	-1.03249	0.31718
C	0.35629	2.66556	1.8171
C	-4.0036	1.52994	0.7369
C	-1.46581	-3.73414	-0.869
O	-2.07543	-2.88778	1.39556
O	2.1533	3.14985	0.40508
C	2.80884	3.37423	-0.83244
H	-1.92818	3.35423	0.49907
H	-2.03998	3.86635	-1.16878
H	-1.62382	1.32388	-1.80694
H	-4.58549	-0.1735	-1.45029
H	-2.90989	-0.50012	-1.88149
H	-3.9043	-2.36817	-0.59616
H	-4.32486	-1.30532	0.7297
H	-1.84296	-0.80601	1.0913
H	0.14445	4.34971	-0.46358
H	0.2393	3.05323	-1.65035
H	0.03716	0.57842	-0.2804
H	0.24213	-1.7049	1.39561
H	0.70314	-3.33661	0.93833
H	2.98187	0.86682	0.45313
H	0.3886	-1.18027	-1.20243
H	1.28313	-2.66689	-1.31709
H	4.3683	-3.35378	-1.18779
H	4.20113	-3.65221	0.53515
H	2.78245	-3.75699	-0.50883
H	5.11364	-1.38799	1.30083
H	5.57161	-1.29199	-0.39312
H	4.71568	0.05522	0.35969
H	0.18861	3.72112	2.06138
H	-0.57405	2.11186	1.97531
H	1.11345	2.26649	2.49778
H	-5.06537	1.54276	0.45396
H	-3.92188	0.85733	1.6008
H	-3.7405	2.53463	1.07417
H	-0.95246	-4.67231	-0.62404
H	-2.52757	-3.96	-0.98958
H	-1.0729	-3.37902	-1.82874
H	2.84277	2.47318	-1.45893
H	2.34684	4.18804	-1.40852
H	3.83264	3.66881	-0.58318

Conf. 3	Coordinates (Angstroms)		
atom	X	Y	Z
C	-2.3879	2.03634	1.144
C	-0.90013	1.92135	1.36974
C	0.06853	2.79233	1.04321
C	1.51699	2.47233	1.37214
C	2.46439	2.411	0.15085
C	2.00669	1.43685	-0.91348
C	-3.10493	0.69501	0.8945

C	-2.79688	-0.08391	-0.41364
C	-1.33511	-0.51478	-0.48226
C	2.57096	0.09513	-1.19659
C	1.70786	-0.92906	-1.92954
C	-0.85454	-1.60886	0.12617
C	1.28494	-2.2169	-1.17797
C	0.54188	-2.12231	0.15169
C	1.03424	-2.63505	1.30909
C	2.36396	-3.33619	1.47604
C	0.27489	-2.57873	2.61841
C	-3.16305	0.75469	-1.64974
C	-0.17516	4.12308	0.37
C	3.7619	-0.44763	-0.435
O	2.87421	1.23411	-2.04053
O	-3.6688	-1.22503	-0.27904
C	-3.77474	-2.09638	-1.39121
H	-2.61502	2.74197	0.33697
H	-2.8521	2.46697	2.04567
H	-0.59412	1.01019	1.88354
H	1.91365	3.23609	2.05849
H	1.56169	1.51631	1.90759
H	3.47549	2.16596	0.49114
H	2.536	3.40204	-0.31745
H	0.96456	1.5848	-1.20582
H	-4.18815	0.86879	0.90397
H	-2.89355	0.01101	1.72582
H	-0.67582	0.15937	-1.01844
H	0.83139	-0.41708	-2.3372
H	2.28838	-1.25697	-2.8041
H	-1.58689	-2.20601	0.66909
H	2.17665	-2.83435	-1.05113
H	0.64191	-2.77201	-1.87602
H	3.00655	-2.79167	2.1823
H	2.20489	-4.33061	1.91744
H	2.92974	-3.47448	0.55417
H	0.91639	-2.16618	3.40949
H	-0.6272	-1.96701	2.5777
H	-0.00901	-3.58812	2.95084
H	-4.22275	1.03127	-1.62241
H	-2.56664	1.67219	-1.68314
H	-2.97026	0.21062	-2.58005
H	0.24228	4.94029	0.97501
H	0.31754	4.18237	-0.60975
H	-1.23662	4.3342	0.22074
H	4.33382	-1.13573	-1.07015
H	4.43458	0.35805	-0.13314
H	3.44484	-0.99524	0.45742
H	-4.33367	-1.64494	-2.22298
H	-2.79266	-2.42265	-1.75825
H	-4.32797	-2.97194	-1.03934

Conf. 4	Coordinates (Angstroms)		
atom	X	Y	Z
C	1.46486	-2.93532	-1.18977
C	1.99022	-1.52422	-1.29073
C	3.05749	-0.98121	-0.67741
C	3.48121	0.44645	-0.98677
C	3.51907	1.41569	0.22027
C	2.16082	1.5887	0.86049
C	-0.06645	-3.10074	-1.06747
C	-0.77933	-2.53115	0.19484
C	-0.92613	-1.0231	0.08453
C	1.19685	2.67618	0.57865
C	-0.28314	2.35704	0.741
C	-2.05938	-0.37544	-0.2376
C	-0.91732	1.8659	-0.58435
C	-2.21005	1.07886	-0.41934
C	-3.43067	1.67645	-0.45299
C	-3.63236	3.16402	-0.63004
C	-4.74787	0.95242	-0.28828
C	-0.0069	-2.90176	1.47399
C	3.95209	-1.7176	0.29392
C	1.52339	3.82931	-0.34978
O	1.96576	2.65761	1.80356
O	-2.06351	-3.18308	0.14402
C	-2.86539	-3.14389	1.31195
H	1.95497	-3.4794	-0.37667
H	1.74978	-3.46858	-2.11075
H	1.44543	-0.89172	-1.99452
H	4.49175	0.42558	-1.42262
H	2.81999	0.86309	-1.75666
H	3.91182	2.3829	-0.10851
H	4.21991	1.04659	0.98064
H	1.72398	0.64627	1.20113
H	-0.28601	-4.17534	-1.07711
H	-0.5617	-2.67372	-1.94766
H	-0.00441	-0.47519	0.25656
H	-0.38819	1.59093	1.51614
H	-0.81884	3.24404	1.10582
H	-2.93906	-0.99062	-0.39502
H	-0.193	1.22355	-1.09862
H	-1.07184	2.72046	-1.24902
H	-4.25698	3.36704	-1.51213
H	-4.17729	3.5787	0.22998
H	-2.70736	3.73261	-0.73473
H	-5.27785	1.3262	0.59953
H	-5.40536	1.15656	-1.14517
H	-4.66026	-0.1291	-0.18437
H	0.11872	-3.98839	1.54753
H	0.98408	-2.43888	1.46777
H	-0.52344	-2.54946	2.37168
H	4.9954	-1.68981	-0.05034
H	3.94304	-1.24995	1.28735
H	3.67543	-2.76619	0.42127
H	1.00551	4.73467	-0.00964
H	2.59374	4.04557	-0.36203
H	1.19717	3.62397	-1.37586
H	-2.48622	-3.81165	2.09832
H	-2.96016	-2.13105	1.72548
H	-3.8569	-3.49539	1.01084

Conf. 5	Coordinates (Angstroms)		
atom	X	Y	Z
C	2.48295	1.94953	-0.15239
C	1.00229	2.21438	0.0199
C	0.41274	2.76993	1.08915
C	-1.07178	3.07335	1.17035
C	-1.95805	2.83417	-0.06362
C	-2.49469	1.42259	-0.23623
C	3.04167	0.69635	0.559
C	2.79376	-0.67043	-0.12614
C	1.3146	-0.96387	-0.31934
C	-2.36655	0.53016	-1.41673
C	-2.66268	-0.95924	-1.26242
C	0.53035	-1.66665	0.51592
C	-1.48272	-1.93948	-1.06551
C	-0.88286	-2.03473	0.33693
C	-1.59785	-2.55317	1.37207
C	-2.98888	-3.12631	1.22515
C	-1.10006	-2.63302	2.79919
C	3.51843	-1.75557	0.69132

C	1.16561	3.18623	2.33248
C	-1.59397	0.92719	-2.65778
O	-3.56377	1.31008	-1.19227
O	3.41055	-0.52225	-1.42392
C	3.433	-1.66929	-2.25251
H	3.05144	2.81135	0.21949
H	2.72002	1.87108	-1.21829
H	0.37273	1.93603	-0.8208
H	-1.5	2.53019	2.02932
H	-1.16699	4.13469	1.44686
H	-1.45707	3.18643	-0.97089
H	-2.85079	3.46412	0.04601
H	-2.71082	0.92081	0.7128
H	2.64362	0.64148	1.57868
H	4.13139	0.80552	0.63836
H	0.90576	-0.52755	-1.22773
H	-3.3789	-1.0643	-0.44255
H	-3.20142	-1.25796	-2.17378
H	0.99694	-2.05445	1.41684
H	-0.6946	-1.71657	-1.7898

H	-1.84536	-2.93615	-1.34912
H	-3.067	-4.06192	1.79566
H	-3.74901	-2.45023	1.64319
H	-3.27173	-3.34265	0.19283
H	-1.8937	-2.31349	3.48794
H	-0.85412	-3.66908	3.07573
H	-0.22798	-2.00994	3.00289
H	4.59703	-1.56805	0.67052
H	3.19423	-1.74127	1.73681
H	3.32842	-2.75866	0.29827
H	0.98306	4.24484	2.56528
H	0.82132	2.61505	3.20656
H	2.24529	3.04241	2.24951
H	-2.11337	0.5535	-3.54883
H	-1.51314	2.01231	-2.75201
H	-0.58328	0.50425	-2.66052
H	4.13323	-2.43542	-1.88956
H	2.43957	-2.12979	-2.35581
H	3.76922	-1.33191	-3.2372

Figure S6g. Re-optimized conformers of (4*S*, 11*R*, 12*R*)-**3** calculated at the B3LYP/6-311G(d,p) level with IEFPCM solvent model for acetonitrile.

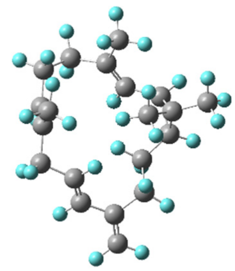

Conf. 1
4.29%
-780.12918558 a. u.

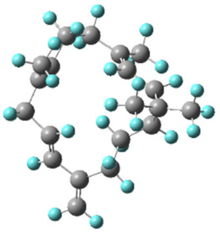

Conf. 2
2.00%
-780.13733449 a. u.

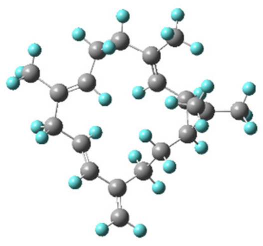

Conf. 3
14.36%
-780.13310085 a. u.

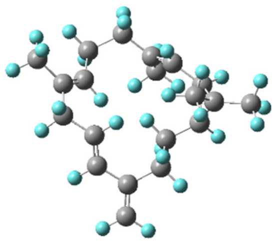

Conf. 4
79.34%
-780.13557317 a. u.

Conf. 1	Coordinates (Angstroms)		
atom	X	Y	Z
C	-2.89044	-0.76114	-1.61945
C	-2.72948	-2.05723	-0.77478
C	-2.46782	0.51826	-0.92405
C	-1.35037	1.15542	-1.30125
C	0.82019	-3.09002	0.60562
C	1.67845	-1.84995	0.59005
C	2.74323	-1.6453	-0.19686

C	3.4903	-0.38038	-0.3276
C	2.81247	0.91378	0.08984
C	1.59188	1.20534	-0.8127
C	-0.74181	2.40198	-0.73756
C	0.76948	2.4381	-0.48706
C	-0.19355	2.53781	0.68089
C	4.7059	-0.3745	-0.89749
C	-0.32819	3.9098	1.32242
C	-0.32833	1.40512	1.6811
H	-1.15837	3.32375	-1.14123
H	1.2597	3.37461	-0.74353

C	-3.38428	1.01018	0.167
C	-1.2978	-2.46829	-0.55169
C	-0.66397	-2.71614	0.60193
C	-1.28489	-2.61456	1.97185
H	-3.947	-0.68365	-1.90489
H	-2.32084	-0.87786	-2.54695
H	-3.23862	-2.85935	-1.32524
H	-3.26707	-1.95347	0.16998
H	-0.80759	0.72074	-2.13964
H	1.03094	-3.68442	1.50419
H	1.06555	-3.71772	-0.25533
H	1.34667	-1.05202	1.24816
H	3.08918	-2.46278	-0.82808
H	2.48994	0.85494	1.13433
H	3.5332	1.73433	0.03038
H	1.94902	1.29536	-1.84628
H	0.94831	0.3267	-0.80219
H	5.17372	-1.29081	-1.24344
H	5.26719	0.54408	-1.02965
H	-1.30493	4.0225	1.8062
H	0.44104	4.05914	2.08838
H	-0.2255	4.70997	0.58471
H	-1.26556	1.49806	2.23805
H	-0.32683	0.42625	1.20427
H	0.48744	1.43807	2.41095
H	-3.0459	1.95477	0.59172
H	-4.39543	1.15683	-0.23113
H	-3.47404	0.28307	0.9809
H	-0.71762	-2.57997	-1.46854
H	-2.30626	-2.23574	1.9563
H	-1.29472	-3.59734	2.45792
H	-0.69163	-1.95771	2.61836

Conf. 2	Coordinates (Angstroms)		
atom	X	Y	Z
C	2.79185	1.6769	-1.17822
C	2.13313	2.57388	-0.11303
C	2.56493	0.17615	-1.12241
C	1.89171	-0.42553	-0.13087
C	-1.76193	2.79115	0.48725
C	-2.23552	1.36574	0.36184
C	-2.96916	0.86465	-0.64052
C	-3.25069	-0.56579	-0.86549
C	-2.32651	-1.59511	-0.23478
C	-0.88604	-1.44628	-0.77665
C	1.59395	-1.87953	0.00385
C	0.16345	-2.40682	-0.24864
C	0.74366	-2.41142	1.15004
C	-4.23898	-0.93602	-1.69513
C	1.14389	-3.76819	1.70889
C	0.22595	-1.46989	2.22303
H	2.38376	-2.54755	-0.32553
H	0.1277	-3.39122	-0.70979
C	3.16003	-0.56522	-2.29467
C	0.62745	2.65539	-0.2108
C	-0.25929	2.80551	0.78132
C	0.09723	2.92458	2.24099
H	3.87542	1.85703	-1.156
H	2.47378	2.02679	-2.17083
H	2.55265	3.57974	-0.24961
H	2.44684	2.25769	0.88557
H	1.49115	0.21273	0.64788
H	-2.28951	3.3026	1.30205
H	-1.9866	3.33762	-0.43281
H	-1.86895	0.69298	1.1327
H	-3.36524	1.54774	-1.39062

H	-2.32048	-1.48435	0.85467
H	-2.7095	-2.59786	-0.44474
H	-0.92134	-1.56201	-1.86694
H	-0.55794	-0.42215	-0.60828
H	-4.87053	-0.20266	-2.1866
H	-4.44505	-1.98038	-1.90271
H	1.92298	-3.66206	2.47199
H	0.28734	-4.26852	2.17421
H	1.53075	-4.4253	0.92566
H	-0.12285	-0.51794	1.82485
H	-0.60936	-1.93038	2.7601
H	1.01038	-1.25468	2.95697
H	3.02196	-1.64413	-2.2268
H	2.70539	-0.22724	-3.2335
H	4.23421	-0.36257	-2.38028
H	0.23417	2.58861	-1.22554
H	1.16326	2.80665	2.43416
H	-0.20665	3.90559	2.62537
H	-0.44224	2.18036	2.83844

Conf. 3	Coordinates (Angstroms)		
atom	X	Y	Z
C	0.49333	-3.06251	-0.64017
C	1.50303	-2.77987	0.51375
C	-0.8605	-2.38844	-0.48416
C	-1.0733	-1.1538	-0.96411
C	3.55114	0.5484	0.00252
C	2.34564	1.38296	-0.31972
C	2.01921	2.55076	0.24812
C	0.82749	3.35812	-0.06179
C	-0.33969	2.7149	-0.78791
C	-1.14887	1.79171	0.15127
C	-2.32893	-0.34114	-0.95769
C	-2.34307	1.11747	-0.50037
C	-3.15372	0.08037	0.25469
C	0.75498	4.62908	0.36582
C	-4.66258	0.1209	0.07436
C	-2.73644	-0.28604	1.66979
H	-2.93751	-0.51196	-1.8457
H	-2.93089	1.78786	-1.12426
C	-1.90853	-3.21365	0.21786
C	2.02041	-1.37068	0.45241
C	3.26298	-0.93738	0.20214
C	4.47752	-1.82041	0.06339
H	0.34403	-4.14567	-0.70352
H	0.95964	-2.75168	-1.57973
H	2.31478	-3.50705	0.44365
H	1.00278	-2.95898	1.47382
H	-0.24457	-0.68885	-1.49706
H	4.07906	0.95451	0.87466
H	4.26533	0.627	-0.83125
H	1.70537	0.97485	-1.09699
H	2.68107	2.96564	1.00717
H	-0.99652	3.49965	-1.17433
H	0.00882	2.14571	-1.65586
H	-0.47785	1.04255	0.57561
H	-1.49907	2.40071	0.99359
H	1.56238	5.08337	0.93117
H	-0.11054	5.24938	0.16004
H	-5.11032	-0.8597	0.27175
H	-5.1176	0.83807	0.76696
H	-4.93727	0.4175	-0.94141
H	-3.27518	-1.17368	2.01311
H	-1.66974	-0.49322	1.75499
H	-2.97978	0.52925	2.36007
H	-2.86614	-2.70068	0.28613

H	-2.0623	-4.15628	-0.32038
H	-1.58822	-3.48445	1.23069
H	1.24622	-0.61612	0.55263
H	4.27588	-2.86528	0.29779
H	4.88103	-1.77128	-0.95485
H	5.2764	-1.47565	0.73023

Conf. 4	Coordinates (Angstroms)		
atom	X	Y	Z
C	1.3532	-2.96762	-0.27965
C	2.55963	-2.0787	-0.7012
C	-0.00588	-2.47155	-0.73342
C	-0.91153	-2.0594	0.16625
C	2.8657	1.50737	0.94147
C	1.45597	1.92076	0.63694
C	1.08512	2.83225	-0.27242
C	-0.2942	3.17429	-0.65903
C	-1.44838	2.30137	-0.19611
C	-1.35847	0.86434	-0.75854
C	-2.285	-1.52917	-0.09851
C	-2.53168	-0.05557	-0.47342
C	-2.94292	-0.57818	0.88911
C	-0.50938	4.22281	-1.47089
C	-4.43356	-0.75755	1.12857
C	-2.18892	-0.16983	2.14275
H	-2.96138	-2.23292	-0.57872
H	-3.37097	0.09104	-1.14933
C	-0.2597	-2.46862	-2.21934
C	2.43044	-0.64896	-0.25253
C	3.18846	0.04854	0.60393
C	4.41504	-0.48007	1.30357
H	1.35524	-3.05838	0.81058
H	1.52741	-3.97293	-0.68518
H	2.6451	-2.09924	-1.79483
H	3.46957	-2.54426	-0.31638
H	-0.61727	-2.1139	1.21261
H	3.05732	1.64718	2.01461
H	3.57786	2.16373	0.42497
H	0.69318	1.38518	1.19523
H	1.86377	3.37089	-0.81131
H	-1.47153	2.26238	0.89782
H	-2.38729	2.76533	-0.51168
H	-1.24443	0.9306	-1.84707
H	-0.44453	0.39775	-0.3988
H	0.30971	4.83538	-1.83403
H	-1.50804	4.49764	-1.79205
H	-4.61748	-1.51842	1.89539
H	-4.89243	0.17702	1.47001
H	-4.94924	-1.0707	0.21698
H	-2.26967	-0.94828	2.90984
H	-1.12752	0.00277	1.96286
H	-2.61154	0.74844	2.56235
H	-1.26323	-2.1187	-2.46226
H	0.45663	-1.82802	-2.7456
H	-0.13651	-3.47651	-2.63306
H	1.58648	-0.12679	-0.69235
H	4.6788	-1.49251	0.99844
H	5.27758	0.16711	1.10559
H	4.27073	-0.47992	2.39018

Figure S6h. Re-optimized conformers of (1*S*, 2*R*)-**4** calculated at the B3LYP/6-311G(d,p) level with IEFPCM solvent model for acetonitrile.
